# Harnessing Supramolecular J‑Aggregates in Deep Eutectic Solvents for Tunable NIR Photothermal and Photodynamic Therapy

**DOI:** 10.1021/acs.nanolett.6c00338

**Published:** 2026-04-24

**Authors:** Krishan Kumar, Meltem Gurol, Pegah Sanjarnia, María Soledad Orellano, Ana Beloqui, Lisa Ebo, Matias L. Picchio, Marcelo Calderón

**Affiliations:** † POLYMAT, Applied Chemistry Department, 652638 University of the Basque Country (UPV/EHU), Paseo Manuel de Lardizábal, 3, 20018 Donostia-San Sebastián, Spain; ‡ Materials Science and Nanotechnology Engineering Department, Engineering Faculty, 52998 Yeditepe University, 26 Ağustos Yerleşimi, 34755 Ataşehir-Istanbul, Turkey; § IKERBASQUE - Basque Foundation for Science, Plaza Euskadi 5, 48009 Bilbao, Spain; ∥ Department of Polymers and Advanced Materials, Faculty of Chemistry, University of the Basque Country (UPV/EHU), Paseo Manuel de Lardizábal, 3, 20018 Donostia-San Sebastián, Spain; ⊥ POLYMAT, Department of Mining-Metallurgy Engineering and Materials Science, School of Engineering, University of the Basque Country (UPV/EHU), Plaza Torres Quevedo 1, 48013 Bilbao, Spain

**Keywords:** Indocyanine green, J-aggregates, NIR, deep eutectic solvent, photothermal therapy, antibacterial properties

## Abstract

J-aggregates of cyanine dyes have emerged as powerful photothermal and imaging agents in the near-infrared (NIR) window due to their red-shifted absorption, narrow bandwidth, and strong excitonic coupling. However, their instability in biological environments often necessitates external carriers or stabilizers that compromise efficiency and biocompatibility. Here, we introduce deep eutectic solvents (DES) as a novel biocompatible medium to enhance the photothermal behavior of J-aggregates under NIR laser irradiation. Under 785 nm laser exposure, J-ICG_DES exhibited rapid, reproducible heating and resistance to disaggregation even at low dye concentrations. Additionally, we observed enhanced *in vitro* reactive oxygen species (ROS) generation for the J-ICG_DES formulation compared to J-ICG_water by employing a singlet oxygen quencher. This synergistic behavior translated into potent antimicrobial efficacy, achieving complete eradication of *Staphylococcus aureus* within 5 min of irradiation.

## Graphical abstract

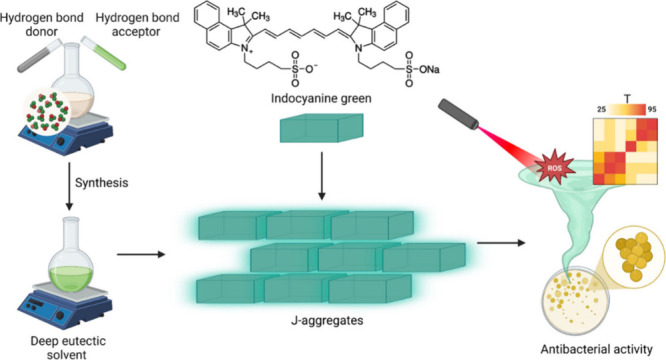

Indocyanine green (ICG), an FDA-approved near-infrared (NIR) dye, has attracted considerable attention in biomedical applications owing to its strong NIR absorption, low cytotoxicity, and deep tissue penetration.^1–4^ Over the past decades, ICG has been widely explored for various clinical and preclinical applications, including fluorescence imaging, photothermal therapy (PTT), and photodynamic therapy (PDT). Its favorable photophysical properties, including its high molar absorptivity and ability to convert absorbed light into heat or reactive oxygen species (ROS), render it particularly attractive for minimally invasive therapeutic strategies.^5,6^ A desirable photothermal agent should exhibit biocompatibility, strong absorption in the NIR region to enable efficient light penetration into deeper tissues, high photostability, and superior photothermal conversion efficiency.^1^ However, the utility of free ICG in biomedical applications is severely limited by several inherent drawbacks such as poor aqueous stability, rapid degradation in physiological environments, low photostability, and strong aggregation in aqueous solutions, leading to diminished quantum yields and unpredictable pharmacokinetics.^7–9^

One strategy to overcome these limitations is to modulate the aggregation behavior of ICG. In aqueous media, ICG readily forms nonemissive H-aggregates, which quench fluorescence and reduce its phototherapeutic efficacy.^10–12^ Conversely, under certain conditions, ICG can form supramolecular assemblies known as J-aggregates, where dye molecules arrange in a head-to-tail configuration, leading to intriguing optical properties such as a very narrow red-shifted absorption band, enhanced light absorption, improved photostability, and extended excited-state lifetimes.^13–16^ J-aggregates of ICG (J-ICG) thus offer significant advantages over free ICG, especially for photothermal and photodynamic applications.^17–21^ However, achieving stable and controllable J-aggregation remains a significant challenge, as the formation and stability of J-ICG are highly dependent on the solvent environment, temperature, ionic strength, and presence of stabilizing agents.

Nanotechnology-based approaches have been extensively explored to address the inherent limitations of J-ICG, particularly its poor hydrolytic stability and suboptimal *in vivo* delivery.^22–28^ Various nanocarrier systems such as polymeric nanoparticles,^22–25^ lipid-based nanoparticles,^26–29^ nanodroplets,^30^ protein nanoparticles,^31^ and other inorganic or organic nanomaterials^32,33^ have been employed to enhance stability, biodistribution, and therapeutic outcomes. However, despite these advancements, the issue of the limited stability of J-ICG in biological milieu remains a significant challenge, often compromising its photothermal and photodynamic therapeutic efficacy over prolonged irradiation.

Recent studies have shown that the use of orthogonal solvent environments, such as perfluorocarbons and hydrocarbons, can promote the supramolecular organization of cyanine dyes by providing distinct microenvironments that stabilize J-aggregates.^4^ In recent years, nonconventional solvents, particularly deep eutectic solvents (DES), have emerged as highly promising media for stabilizing functional molecules and nanomaterials.^34–40^ Notably, molecular aggregation phenomena are not limited to synthetic dye systems; recent findings have demonstrated that biological macromolecules such as extracellular RNA can form nucleic acid–protein aggregates through electrostatic interactions, underscoring the universal relevance of controlled molecular assembly across both synthetic and biological contexts.^41^ DES are typically composed of a mixture of a hydrogen bond acceptor (HBA) and a hydrogen bond donor (HBD), which interact via extensive hydrogen bonding to form a eutectic mixture showing an abnormally large depression in its melting point. Owing to their unique physicochemical properties, such as high thermal and chemical stability, low volatility, tunable polarity, and excellent biocompatibility, DES have been extensively explored in diverse fields, including extraction, catalysis, electrochemistry, and more recently, biomedical sciences.^36^

The ability of DES to provide tailored microenvironments through adjustable hydrogen bonding networks makes them particularly attractive for modulating aggregation processes and stabilizing supramolecular assemblies such as J-aggregates.^35^ The polarity, viscosity, and hydrogen bonding capability of DES can profoundly influence the self-assembly process, molecular orientation, and photophysical behavior of chromophores, overcoming several of the limitations associated with nanocarriers. Moreover, many DES components are derived from natural, biocompatible substances, making them suitable for biomedical applications where traditional organic solvents would be toxic or incompatible.^37^ Additionally, the versatile chemical design of DES allows fine-tuning of solvent polarity, viscosity, and hydrogen bond density, offering a new route to stabilize and modulate ICG-based phototherapeutics without the complexity and scalability issues often associated with nanocarrier systems.

Despite the promising potential of DES in stabilizing functional aggregates, no systematic studies have evaluated their role in the stabilization and photophysical modulation of J-ICG. In this context, we aim to systematically investigate the stabilization and photophysical tuning of J-ICG in two distinct sets of biocompatible DES to understand the influence of different hydrogen bond donor groups. The hydrophilic DES series utilized choline chloride (ChCl) as the HBA combined with various HBDs such as urea (U), glycerol (Gly), pyrogallol (Py), glycolic acid (GlyAc), tannic acid (TA), citric acid (CA), and sorbitol (Sor), enabling the generation of highly polar environments with varying hydrogen bonding densities. In contrast, the hydrophobic DES series employed menthol (Men) as the HBA, paired with a range of HBDs such as oleic acid (OleAc), dodecanoic acid (DodAc), thymol (Thy), lidocaine (Lid), eucalyptol (Euc), and ibuprofen (Ibu), generating less polar and nonionic microenvironments.

To the best of our knowledge, this is the first report on the modulation of J-ICG photothermal behavior using DES. Under this scenario, we hypothesized that the J-ICG/ICG equilibrium could be confined in a modulating environment (hydrophilic/hydrophobic). Moreover, this confined state can act as a reservoir continuously replenishing active monomers and thereby sustaining photothermal and ROS generation under NIR irradiation. Building on this framework, we evaluated the suitability of selected J-ICG_DES formulations for photothermal and photodynamic applications, specifically investigating their potential to induce antibacterial effects against *Staphylococcus aureus* under irradiation conditions relevant to biomedical use (Figure 1a). Such a strategy highlights the potential of DES to function as a protective and performance-enhancing medium for J-ICG, offering a basis for the future development of DES-based platforms in biomedicine.

**Figure 1:**
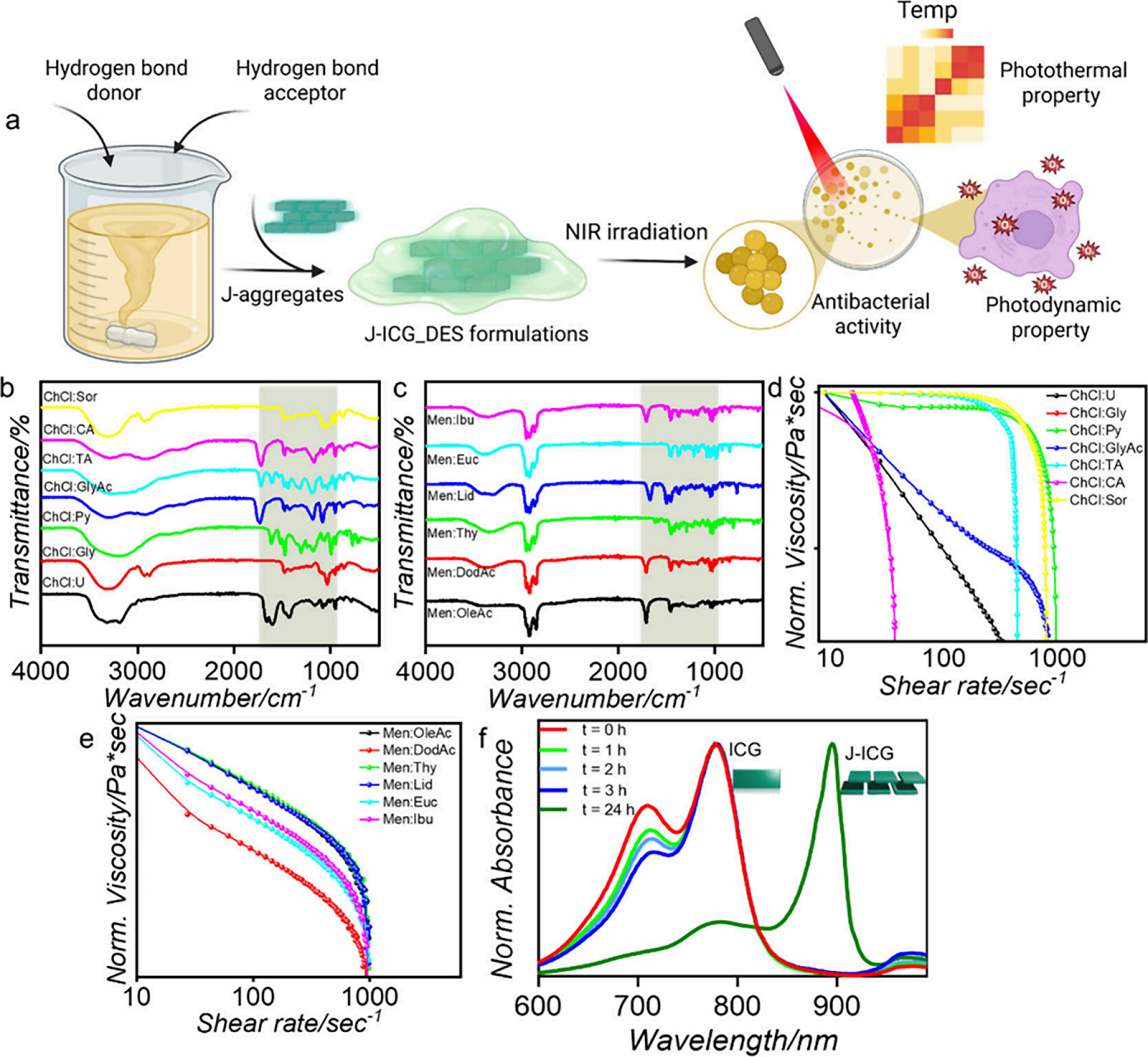
(a) Schematic illustration of the J-ICG modulation using DES formulations. ICG forms stabilized J-aggregates within the DES matrix, which under 785 nm irradiation display enhanced photothermal heating and ROS generation. The resulting photothermal/photodynamic effects were evaluated for antibacterial activity against *Staphylococcus aureus*. Physicochemical characterization of DES. (b, c) FTIR spectra highlighting the characteristic functional groups and intermolecular interactions, including hydrogen bonding, between the DES components, indicating successful formation of the eutectic mixture. (d, e) Normalized viscosity measurements of the DES formulations, demonstrating their rheological behavior and the influence of composition on fluidity under controlled experimental conditions. (f) Time-dependent UV–vis spectra illustrating the formation of J-aggregates, as evidenced by a progressive red-shift in the absorption peak over time.

## Synthesis and Characterization of DES and J-ICG

The DES were prepared by simply combining the HBD and HBA components in appropriate molar ratios. The mixtures were placed in 26 mL glass vials and stirred at 200 rpm on a magnetic stirrer at 90 °C until a homogeneous transparent liquid was obtained. The specific molar ratios used for the preparation of each DES formulation are summarized in Table 1. The molar ratios were chosen to correspond to the eutectic composition of each HBD–HBA pair, which varies due to differences in hydrogen bonding interactions and molecular structures. J-ICG was prepared as a concentrated stock solution at 10 mg mL^–1^ (see the Experimental Section in the Supporting Information for details). For the preparation of the photothermal formulations, 2.5 μL of the J-ICG stock was added to 97.5 μL of the DES, yielding a final volume of 100 μL without the addition of water. The mixture was briefly vortexed and allowed to equilibrate for 5–10 min to ensure complete dispersion and stabilization of the J-aggregates within the DES. All samples were freshly prepared immediately before photophysical and ROS generation experiments.

**Table 1:** Compositions and Molar Ratios Used for the Synthesis of Hydrophilic and Hydrophobic DES^a^

HBA	HBD	Molar ratio
Hydrophilic DES		
ChCl	U	1:2
ChCl	Gly	1:2
ChCl	Py	1:2
ChCl	GlyAc	1:2
ChCl	TA	20:1
ChCl	CA	1:1
ChCl	Sor	1:1
Hydrophobic DES		
Men	OleAc	2:1
Men	DodAc	3:1
Men	Thy	2:1
Men	Lid	2:1
Men	Euc	1:1
Men	Ibu	3:1

a Hydrophilic DES were prepared by using ChCl as the HBA, combined with various HBDs. Hydrophobic DES were prepared using Men as the HBA with different HBDs. The specific molar ratios employed for each combination are listed.

Hydrophilic and hydrophobic DES systems were compared to elucidate the influence of solvent polarity, hydrogen bonding environment, and water miscibility on the stabilization and photothermal response of ICG/J-ICG. Furthermore, systematic variation of the HBD:HBA molar ratio was employed to probe the effect of the hydrogen bonding density and deviation from the eutectic composition on the structural and physicochemical characteristics of the DES, thereby providing insight into their role in modulating dye–solvent interactions. The physicochemical characterization of the DES was conducted to confirm their successful formation and evaluate their structural and rheological properties. The FTIR spectra (Figure 1b,c) reveal notable shifts and broadening in the characteristic absorption bands associated with functional groups of the individual components upon DES formation. Specifically, the broadening of the O–H stretching vibrations indicates extensive hydrogen bonding interactions between hydrogen bond donors and acceptors, which are essential for stabilization of the eutectic mixture. Additional shifts in the C=O and N–H stretching regions further support the occurrence of molecular interactions characteristic of DES formation. These spectral changes confirm that the components do not merely form physical mixtures but engage in strong noncovalent interactions, leading to a stable DES network.

The viscosity data demonstrate that the formulations exhibit composition-dependent variations in fluidity (Figure 1d,e). The observed viscosities reflect the balance between molecular interactions (such as hydrogen bonding) and the steric properties of the individual components. The Newtonian viscosity (*n*_0_) was also determined for all DES, which is shown in Table S1. Systems with stronger intermolecular interactions generally exhibit higher viscosities; for instance, ChCl:TA (45.1 Pa·s) and ChCl:CA (910.3 Pa·s) show ∼1.57- and ∼31.7-fold higher viscosities than ChCl:Sor (28.7 Pa·s), consistent with the formation of more structured hydrogen bonding networks. The menthol-based DES feature lower viscosities than ChCl-based systems, as the absence of ionic interactions reduces internal friction within the solvent matrix. Time-resolved UV–vis spectroscopy was employed to verify the formation of the ICG J-aggregates. As shown in Figure 1f, a progressive red-shift in the absorption maximum (Δλ_J‑ICG_ ∼ 100 nm) relative to that of monomeric ICG in aqueous solution was observed over 24 h, providing strong evidence for the development of stable J-aggregates.

## Photothermal Performance of J-ICG/ICG in the Presence of DES

The photothermal performance of J-ICG was systematically evaluated in the presence of various hydrophilic and hydrophobic DES by using repetitive heating–cooling cycles (Figure 2). Although J-aggregated ICG exhibits a red-shifted absorption maximum (∼890–900 nm), the aggregates retain appreciable absorption at 785 nm. In addition, a dynamic equilibrium between J-ICG and residual monomeric ICG ensures that both species contribute to excitation and subsequent heat generation under 785 nm irradiation.^34^ This approach allows for the reliable comparison of photothermal responses across different DES formulations under identical irradiation conditions. The results reveal a clear dependence of the maximum temperature (*T*_max_) on the chemical composition of the DES formulations (Figures S1 and S2, Tables S2 and S3). UV–vis absorption spectra of J-ICG_DES were also recorded across the 550–950 nm range, which confirm that the characteristic J-aggregate is preserved across the majority of DES formulations (Figures S3 and S4). The thermal stability and stability in biologically relevant media of the J-ICG_DES system are systematically demonstrated in Figures S5–S8. The ChCl:Gly system exhibited the highest *T*_max_, reaching 96.4 ± 6.7 °C, a ∼1.23-fold increase (∼23%) compared to that of J-ICG_water (78.5 ± 2.8 °C). Other DES such as ChCl:U and ChCl:TA result in ∼7 and ∼11% decreases compared to aqueous medium (Figure 2a). The enhanced photothermal response in ChCl:Gly may be attributed to the strong and extensive hydrogen bonding between ChCl and Gly, which likely stabilizes the J-ICG aggregates and promotes efficient nonradiative relaxation pathways. In contrast, DES formulations such as ChCl:GlyAc (∼64%), ChCl:CA (∼50%), and ChCl:Py (∼31%) demonstrated a reduction in the *T*_max_ value, suggesting weaker hydrogen bonding interactions or less favorable microenvironments for effective photothermal conversion. ChCl:Sor also demonstrated moderate heating behavior, reaching 59.6 ± 2.1 °C (∼24% reduction), likely due to its multiple hydroxyl groups contributing to moderate hydrogen bonding networks.

**Figure 2:**
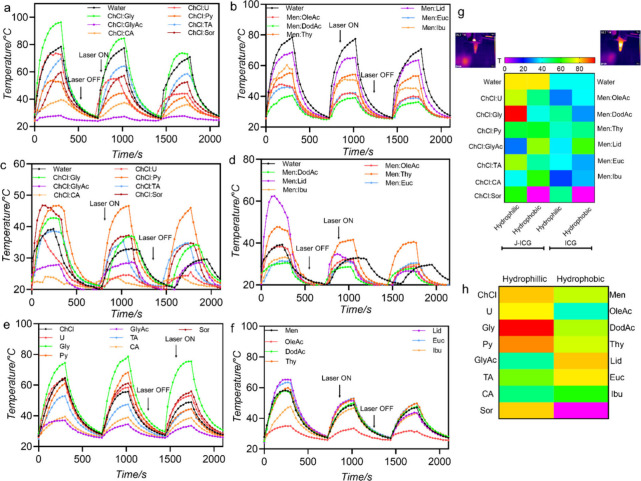
Photothermal behavior and aggregation dynamics of J-ICG in the presence of various DES formulations. Heating–cooling cycles (5 min heating, 7 min cooling) of J-ICG in (a) hydrophilic and (b) hydrophobic DES; ICG monomer in (c) hydrophilic and (d) hydrophobic DES; and J-ICG in the presence of individual components of (e) hydrophilic and (f) hydrophobic DES, demonstrating distinct thermal responses that are dependent on the solvent. (g) Infrared thermal images (inset) show the surface temperature of the samples after heating. (g, h) The corresponding heat maps summarize the maximum temperatures (*T*_max_) achieved during the heating cycles for each DES system, emphasizing the role of hydrogen bonding interactions in modulating the photothermal behavior of J-ICG and ICG monomer.

In the hydrophobic DES systems based on menthol, overall lower *T*_max_ values were recorded compared to the hydrophilic series as well as J-ICG_water, indicating a reduced capacity for thermal conversion (Figure 2b). Among these, Men:Lid exhibited the highest *T*_max_ of 68.7 ± 1.5 °C, representing an increase of ∼70%, followed by Men:Ibu (60.5 ± 3.8 °C, ∼49%) and Men:Thy (55.2 ± 0.4 °C, ∼36%), and formulations such as Men:OleAc (47.9 ± 1.9 °C, ∼18%) and Men:Euc (45.7 ± 1.7 °C, ∼13%) showed a more moderate heating performance relative to Men:DodAc (40.5 ± 0.9 °C). The generally reduced photothermal efficiency in hydrophobic DES may result from the limited hydrogen bonding capabilities of menthol and the greater hydrophobic environment, which could restrict the aggregation and energy dissipation behavior of J-ICG.

The heating–cooling profiles for ICG monomers were also studied in order to see the photophysical properties in the presence of two DES environments (Figure 2c,d,h and Figure S2). During the heating cycles, certain DES systems induced higher *T*_max_ values, indicating enhanced photothermal conversion efficiency, while others showed more moderate temperature rises. Notably, the temperature peaks are sharper and higher in ChCl-based formulations compared to those of Men-based formulations, suggesting stronger interactions between ICG and the solvent matrix. Among the formulations, ChCl:Sor (46.8 ± 0.4 °C, ∼19%), ChCl:Gly (46.7 ± 0.7 °C, ∼19%), and ChCl:Py (42.8 ± 1.1 °C, ∼9%) displayed enhanced *T*_max_ values, whereas ChCl:U (28.7 °C, ∼27%), ChCl:CA (24.0 ± 0.1 °C, ∼39%), ChCl:TA (38.7 ± 0.2 °C, ∼2%), and ChCl:GlyAc (37.3 ± 0.1 °C, ∼5%) exhibited decreased *T*_max_ values, compared with that of ICG_water (39.3 ± 1.3 °C). The photothermal performance of ICG varied considerably across Men-based DES compared with that of ICG_water (39.3 °C). Men:Lid showed the highest enhancement, reaching 62.5 ± 1.4 °C (∼59%), followed by Men:Thy at 47.7 ± 1.1 °C (∼21%). By contrast, Men:OleAc (38.4 ± 0.3 °C, ∼2%), Men:Euc (32.1 ± 0.1 °C, ∼18%), Men:Ibu (33.3 ± 0.4 °C, ∼15%), and Men:DodAc (30.5 ± 0.2 °C, ∼22%) all exhibited reduced *T*_max_ relative to that of water. These results suggest that while certain DES components (e.g., Lid and Thy) can enhance the photothermal conversion of ICG, others, particularly fatty acid- and alcohol-based systems, provide less favorable environments for efficient heating. During the cooling cycles, all systems exhibit gradual relaxation to baseline temperatures, but the rate of temperature decay varies, reflecting differences in the thermal dissipation properties and viscosity between the DES. Systems with a higher *T*_max_ generally retain heat longer, indicating a combination of efficient light absorption and slower heat loss.

In ChCl-based DES, both J-ICG and ICG show the highest photothermal performance in ChCl:Gly, with an increase of ∼23% (J-ICG) and ∼19% (ICG), indicating that strong hydrogen bonding stabilizes both aggregates and monomers. In contrast, ChCl:Py, ChCl:GlyAc, and ChCl:CA exhibit opposite trends, where J-ICG decreases sharply (∼31%, ∼64%, and ∼50%) and ICG shows smaller decreases of ∼5% (ChCl:GlyAc) and ∼39% (ChCl:CA), reflecting the higher sensitivity of J-ICG aggregates to the solvent environment. ChCl:Sor enhances ICG (∼19%) but reduces J-ICG (∼31%), further highlighting the differential effects on monomers vs aggregates. In Men-based DES, J-ICG shows a lower *T*_max_ compared to water and ChCl-based DES, whereas for ICG, a substantial increase was observed for Men:Lid (∼59%), and Men:Thy (∼21%).

Nevertheless, the heating performance varied across DES compositions, underscoring that specific molecular interactions and medium viscosity directly govern the photothermal outcomes. To probe this effect, the individual DES components were evaluated separately, and UV–vis spectra were recorded at the end of each irradiation cycle (Figures S9 and S10). Notably, their behavior differed markedly from that of the complete DES, highlighting the critical role of intermolecular hydrogen bonding networks in enhancing photothermal performance (Figure 2e,f,h; Tables S4 and S5). Another possible contribution is the altered photothermal response under 785 nm irradiation arising from residual monomeric ICG or the gradual release of ICG from J-ICG depots. However, the superior performance observed in DES compared to ICG or J-ICG in water indicates that DES act as stabilizing and modulatory microenvironments, favoring aggregate stabilization and facilitating more efficient nonradiative energy dissipation, thereby yielding enhanced photothermal effects. Thermal stability analysis at 95 °C further demonstrated that ChCl:Gly and ChCl:Sor uniquely preserve the J-ICG over 8 min, in stark contrast to water, where complete J-aggregate dissociation occurs within 5 min (Figures S5–S7).

The comparative analysis (Figure 3) between Newtonian viscosity and *T*_max_ under NIR laser irradiation demonstrates an inverse relationship between the viscosity and photothermal performance of J-ICG in both ChCl- and Men-based DES systems. In the ChCl-based DES series, lower viscosity solvents such as ChCl:U (0.1146 Pa·s) and ChCl:Gly (0.2973 Pa·s) facilitated higher *T*_max_ values, attributed to their moderate hydrogen bonding networks that allow both efficient molecular mobility and stable J-aggregate formation. Conversely, highly viscous systems such as ChCl:CA (910.3070 Pa·s) and ChCl:TA (45.0546 Pa·s), dominated by multidentate polyhydroxy carboxylic acid structures, exhibited significantly reduced *T*_max_, likely due to restricted molecular diffusion and aggregation dynamics within the rigid hydrogen-bonded networks. A similar but less pronounced trend was observed in Men-based DES, where lower viscosity systems such as Men:OleAc (0.1647 Pa·s), Men:Thy (0.1862 Pa·s), and Men:Lid (0.1986 Pa·s) displayed higher *T*_max_, reflecting more favorable conditions for aggregate stability and heat dissipation. The relatively higher viscosity of Men:DodAc (1.67200 Pa·s), driven by its long alkyl chain and van der Waals interactions, corresponded to lower *T*_max_.

**Figure 3:**
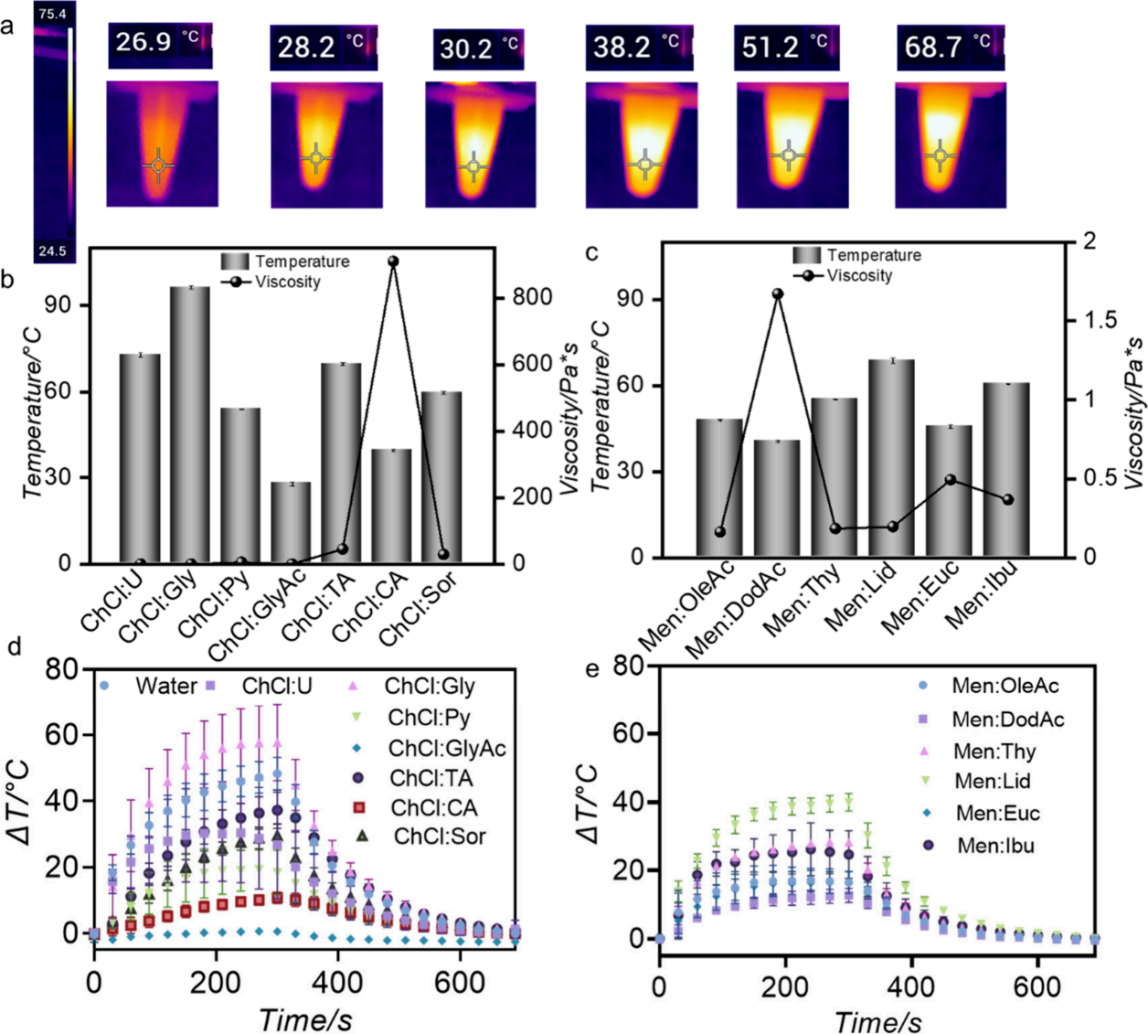
Photothermal conversion efficiency and mechanism. (a) Infrared thermal images at time 0, 6, 8, 25, 66, and 273 s, respectively. Correlation between *T*_max_ achieved during photothermal heating of the solution containing J-ICG under the NIR laser and Newtonian viscosity (*n*_0_) of (a) hydrophilic and (b) hydrophobic DES. Bar graphs represent the maximum temperature reached during the heating cycles, while the overlaid line plots depict the corresponding *n*_0_ values for each DES formulation. Steady-state heating curve for J-ICG in the presence of (d) hydrophilic and (e) hydrophobic DES. Data are presented as mean ± SD (*n* = 3).

In contrast, ChCl-based DES with highly viscous or sterically voluminous donors (such as CA, Sor, or TA) limited molecular mobility and aggregate formation, resulting in diminished photothermal responses. Similarly, among Men-based DES, Lid and Ibu, which possess amide and carboxyl functionalities capable of forming strong directional hydrogen bonds with Men, supported better aggregate stabilization and superior photothermal performance. Conversely, fatty acid-based DES such as oleic acid and dodecanoic acid, characterized by long hydrophobic chains and weaker hydrogen bonding interactions, were less effective in stabilizing J-aggregates and yielded lower photothermal efficiencies.

The photothermal conversion efficiency (η) of J-ICG varied dramatically depending on the DES composition (Figure 3d,e; Tables S6 and S7). In the ChCl-based (hydrophilic) series, ChCl:Gly achieved the highest η (21.7%), nearly doubling the efficiency of water (15.4%), followed by ChCl:TA (16.2%) and ChCl:U (12.6%), confirming the importance of strong hydrogen bonding networks in stabilizing aggregates and promoting efficient nonradiative decay. In contrast, several ChCl-based DES significantly suppressed performance, with ChCl:Py (7.1%), ChCl:CA (3.4%), and ChCl:Sor (9.0%) displaying poor efficiencies, suggesting the destabilization of aggregates or unfavorable microenvironments for heat dissipation. In the menthol-based (hydrophobic) series, efficiencies were uniformly low, with the best systems (Men:Lid (12.6%), Men:Thy (9.6%), and Men:Ibu (9.5%)) remaining an order of magnitude below ChCl:Gly. Men:OleAc (8.0%), Men:Euc (6.9%), and Men:DodAc (5.7%) further underscored the poor compatibility of hydrophobic DES for sustaining J-ICG aggregation and thermal conversion. Collectively, these findings establish that DES are not universally beneficial; rather, the chemical identity of the hydrogen bond donor is decisive, with hydrophilic systems (notably ChCl:Gly) acting as powerful enhancers, while hydrophobic DES substantially limit photothermal efficiency. ATR-FTIR analysis of J-ICG across hydrophilic DES formulations reveals systematic shifts and broadening of C–O stretching bands (∼900–1200 cm^–1^), confirming differential hydrogen bonding interactions between the DES matrix and J-ICG (Figure S11). Despite possessing a greater number of hydroxyl groups, ChCl:Sor induces more pronounced spectral shifts yet demonstrates inferior photothermal performance compared to ChCl:Gly, a finding attributed to its nearly 100-fold higher viscosity (28.67 vs 0.30 Pa·s) and excessively strong hydrogen bonding environment that overconstrains the J-aggregate molecular packing geometry, disrupting the precise intermolecular distances required for optimal exciton coupling. These observations collectively demonstrate that photothermal performance is governed by a multiparameter microenvironmental balance with ChCl:Gly uniquely providing the optimal combination of hydrogen bonding organization, molecular fluidity, and J-aggregate packing geometry required for maximum photothermal conversion efficiency.

## *In Vitro* ROS Generation and Antimicrobial Properties

The ability of J-ICG/ICG to generate ROS upon irradiation was evaluated by using DPBF as a chemical probe. DPBF serves as a well-established singlet oxygen scavenger, and its characteristic absorbance decrease correlates directly with ROS production (Figure 4a,b and Figure S12). The results obtained for J-ICG in water and the ChCl:Gly DES system revealed significant differences in ROS generation efficiency as a function of irradiation time. In aqueous solution (Figure S12a), almost no difference in DPBF absorbance with increasing irradiation time was observed, underscoring the inability to sustain ROS production. In contrast, J-ICG_ChCl:Gly displayed rapid and extensive bleaching (∼57% at 20 min), indicating robust singlet oxygen generation facilitated by the strong hydrogen bonding network of the DES that stabilizes aggregates and promotes efficient energy transfer. For the ICG monomer in ChCl:Gly, there is a considerably lower generation of ROS, as ∼22% bleaching was observed (Figure 4b and Figure S12b). This indicates that monomeric ICG is a weaker ^1^O_2_ producer, and the effect of the eutectic solvent on the singlet oxygen yield is far more pronounced for the J-ICG species than for the monomer. These results demonstrate that DES not only stabilize J-ICG aggregates but also endow them with superior ROS-generating capacity compared to ICG monomers, highlighting the essential role of the solvent microenvironment in dictating photodynamic outcomes. These effects can be due to enhanced intersystem crossing in DES, which facilitate triplet-state formation, promoting singlet oxygen production.^32^ Furthermore, the increased viscosity of the DES may reduce nonradiative relaxation pathways, prolonging the excited-state lifetimes, promoting more efficient energy transfer to molecular oxygen, and favoring type II (singlet oxygen) photochemistry.^32^ The superior ROS-generating capacity of J-ICG_DES is attributed to two synergistic mechanisms: The rigid, structured DES microenvironment restricts intramolecular motion of the ICG polymethine chain, reducing the energy gap of the intersystem crossing process and facilitating more efficient triplet-state population and subsequent ^1^O_2_ generation via energy transfer to molecular oxygen.^42^ Concurrently, the elevated viscosity of the DES matrix, particularly ChCl:Gly, suppresses diffusion-mediated triplet-state quenching pathways, extending the effective triplet-state lifetime and increasing the probability of productive ^3^O_2_ sensitization, consistent with the restricted intramolecular motion framework established for aggregation-induced intersystem crossing in organic photosensitizers.^43,44^ To further characterize the ROS profile of the J-ICG_DES system, terephthalic acid fluorescence assays were also performed, and results revealed negligible hydroxyl radical generation under NIR irradiation, indicating that the photodynamic activity is predominantly mediated by singlet oxygen (Figure S13). Complementary EPR spin-trapping experiments using phenyl *N*-*t*-butylnitrone confirmed the generation of radical species exclusively upon NIR irradiation, with hyperfine coupling parameters (AN = 15.89 G, AH = 3.52 G) consistent with the formation of •OOH (Figure S14).

**Figure 4:**
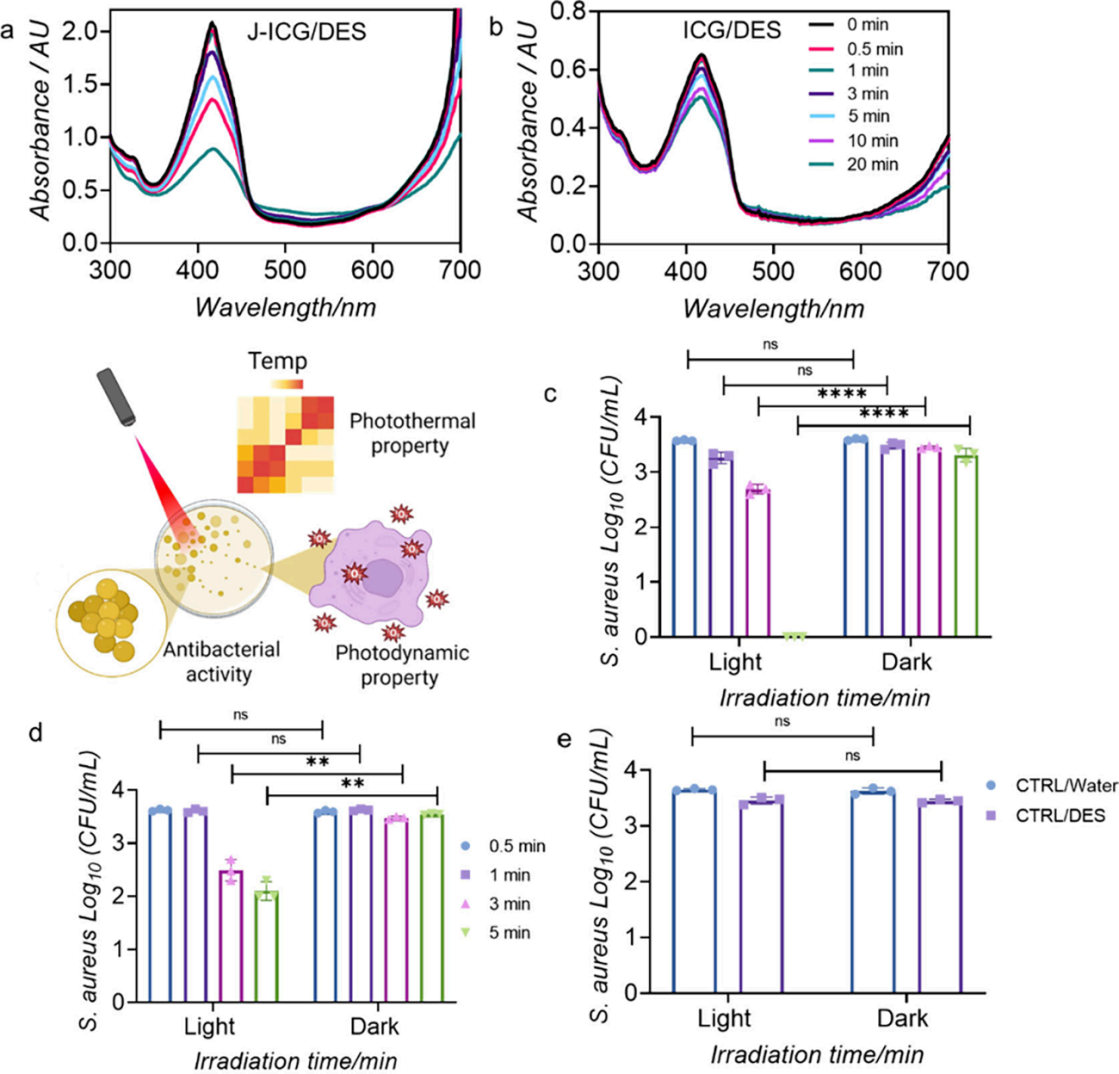
Detection of ROS generation by J-ICG using 1,3-diphenyl­iso­benzo­furan (DPBF) as an external probe. (a) J-ICG and (b) ICG monomer in ChCl:Gly DES system, showing the progressive decrease in DPBF absorbance with increasing irradiation time, indicating ROS production. Antibacterial activity of J-ICG against *S. aureus* was studied under laser ON/OFF conditions. CFU/mL of MRSA 43300 after incubation with J-ICG in (c) DES, (d) water, and (e) controls under NIR irradiation or in the dark. Results are expressed as the mean ± standard deviation from three independent experiments (*n* = 3). Statistical analysis was performed using two-way ANOVA followed by Bonferroni’s multiple comparison test. Asterisks denote significant differences (*p < 0.05, **p < 0.01, ***p < 0.001, ****p < 0.0001).

The antimicrobial activity of J-ICG/ICG_DES formulations against *S. aureus* was evaluated under NIR laser irradiation and in the dark to assess their efficacy via combined photothermal and photodynamic mechanisms. Colony-forming unit (CFU) counting was performed for samples exposed to NIR irradiation at different times to determine the effect of the different conditions on bacterial viability. As shown in Figure 4c,d J-ICG_DES and J-ICG in water exhibited a time-dependent CFU reduction, with complete bacterial killing achieved only in DES after 5 min of irradiation. A similar behavior was observed for monomeric ICG (Figure S15). In contrast, no reduction in bacterial viability was observed under dark conditions, confirming that the strong antimicrobial activity observed for J-ICG in DES occurs specifically upon NIR irradiation. The antimicrobial effect of J-ICG can be attributed to the combined action of photothermal activity and ROS generation since J-ICG in DES exhibited higher photothermal conversion and ROS generation capacity compared to that in water (Figures 2 and 4). To exclude possible solvent-related effects, the impact of DES and water alone (without ICG monomers or aggregates) was evaluated after 5 min of irradiation (Figure 4e). No reduction in bacterial viability was observed, supporting the idea that the bactericidal effect comes exclusively from ICG monomers or aggregates in DES upon NIR irradiation. Figure S16 shows the percentage reduction in bacterial viability under NIR irradiation, with J-ICG in ChCl:Gly achieving complete (100%) killing within 5 min.

The performance of the ICG monomer and J-ICG dispersed in DES highlights the critical role of hydrogen bonding networks and DES composition in modulating J-aggregate stability and heat generation. In general, the ICG monomer exhibited significantly lower temperature elevation across both ChCl-based and Men-based DES, confirming the limited photothermal capacity of monomeric ICG due to its lower absorption cross-section and poor photothermal conversion due to photobleaching. Upon the formation of J-aggregates, efficiency was modulated when J-ICG was incorporated into DES, where the nature of the HBD played a pivotal role. ChCl-based DES containing polyhydroxylated donors, such as glycerol and urea, provided optimal hydrogen bonding environments that stabilized J-ICG aggregates and promoted efficient heat conversion, as reflected by the highest *T*_max_. Taken together, these results highlight that DES provides confined, highly tunable microenvironments in which J-ICG molecules can be stabilized through specific hydrogen bonding interactions and reduced molecular mobility.

The present study highlights the critical role of DES as a versatile class of solvent systems for stabilizing functional supramolecular assemblies in biologically compatible environments. Within DES, stable J-aggregate formation was achieved, with ChCl:Gly proving especially effective in preserving J-ICG aggregates. This stabilization not only enhanced the photothermal and photodynamic efficiency but also mitigated degradation by maintaining a dynamic J-ICG/ICG equilibrium that continuously replenishes active monomers. Thus, DES provide a unique solvent microenvironment that can act as a functional surrogate for conventional nanocarriers, offering both improved performance and potential biocompatibility. Importantly, ROS generation was markedly enhanced in DES relative to aqueous media, as confirmed by accelerated DPBF bleaching in ChCl:Gly. Together, these findings emphasize the dual role of DES as both stabilizing media and functional modulators that fine-tune photothermal and photodynamic responses. The intrinsic tunability of DES through the control of viscosity, hydrogen bonding, and polarity positions them as adaptable platforms for advanced phototherapeutic applications, including cancer therapy, imaging, and antimicrobial treatments.

## Supplementary Material



## Notes

Experimental section; instrumentation (UV–vis spectroscopy, FTIR, rheometer, NIR laser, photothermal camera, and EPR); methods and discussion (FTIR spectra and viscosity measurements of hydrophilic and hydrophobic DES, NIR laser irradiation of J-ICG in hydrophilic, hydrophobic, and individual components of DES, NIR laser irradiation of ICG monomer in the presence of DES, UV–visible spectra of J-ICG at the end of each irradiating cycle, and photothermal efficiency of J-ICG in hydrophilic and hydrophobic DES); and *in vitro* ROS detection and antimicrobial activity (PDF)
