# Supplementary material for: Harnessing Supramolecular J‑Aggregates in Deep Eutectic Solvents for Tunable NIR Photothermal and Photodynamic Therapy
Source: Nano Lett. 2026 Apr 24;26(17):5747–56. doi: 10.1021/acs.nanolett.6c00338 (PMC13154349; doi:10.1021/acs.nanolett.6c00338)
Supplement: Supplementary file 1 [file nl6c00338_si_001.pdf]

Supporting Information

Harnessing Supramolecular J-Aggregates in Deep Eutectic Solvents for Tunable NIR Photothermal and Photodynamic Therapy

Krishan Kumar,*[a] Meltem Gurol,[a,b] Pegah Sanjarnia,[a] María Soledad Orellano,[a] Ana Beloqui,[a,c] Lisa Ebo,[d] Matias L. Picchio,*[c,e] Marcelo Calderon*[a,c]

[a]POLYMAT, Applied Chemistry Department, University of the Basque Country (UPV/EHU), Paseo Manuel de Lardizábal, 3, 20018, Donostia-San Sebastián, Spain.

[b]Materials Science and Nanotechnology Engineering Department, Engineering Faculty, Yeditepe University, 26 Ağustos Yerleşimi, 34755 Ataşehir-Istanbul, Turkey.

[c]IKERBASQUE - Basque Foundation for Science, Plaza Euskadi 5, 48009 Bilbao, Spain.

[d]Department of Polymers and Advanced Materials, Faculty of Chemistry, University of the Basque Country (UPV/EHU), Paseo Manuel de Lardizábal, 3, 20018, Donostia-San Sebastián, Spain.

[e]POLYMAT, Department of Mining-Metallurgy Engineering and Materials Science, School of Engineering, University of the Basque Country (UPV/EHU), Plaza Torres Quevedo 1, 48013 Bilbao, Spain.

E-mail: krishan.kumar@ehu.eus, matiasluis.picchiop@ehu.eus, marcelo.calderonc@ehu.eus

Index

S1. Experimental Section **S3**
S1.1 Methods S3
S2.2 Materials S4
S2. Instrumentation **S5**
S2.1 UV/Vis spectroscopy S5
S2.2 FTIR S5
S2.3 Rheometer S5
S2.4 NIR Laser S6
S2.5 Photothermal Camera S6
S2.6 Electron paramagentic resonance (EPR) S6
S3. Methods and Discussion **S7**
S3.1 FTIR spectra of hydrophilic and hydrophobic DES S7
S3.2 Viscosity measurements of hydrophilic and hydrophobic DES S8
S3.3 Near infrared-laser irradiation S9
S3.3.1 NIR-laser irradiation of J-ICG in hydrophilic and hydrophobic DES S9
S3.3.2 NIR-laser irradiation of J-ICG in individual components of DES S12
S3.3.3 NIR-laser irradiation of ICG monomer in DES S12
S3.3.4 UV-visible spectra of J-ICG at end of each irradiating cycle S13
S3.3.5 Thermal and biological stability of J-ICG in presence of DES S14
S3.3.6 FTIR Spectra of J-ICG_DES formulation S18
S3.4 Photothermal conversion efficiency S19
S3.4.1 Photothermal efficiency of J-ICG in hydrophilic DES S19
S3.4.2 Photothermal efficiency of J-ICG in hydrophobic DES S20
S4. *In vitro* ROS detection and antimicrobial activity **S20**

S1. Experimental Section

S1.1 Methods

Preparation of ICG J-aggregates: J-ICG was prepared according to a previously reported protocol.[1] In brief, an aqueous solution of ICG (10 mg mL^{-1}) was incubated at 80 °C for 20 h, followed by dialysis against water for 24 h to eliminate unbound dye. The formation of J-aggregates was monitored by UV-Vis spectroscopy using a Cary 100 Bio spectrophotometer (Agilent). The resulting J-ICG solution was stored at 4 °C until further use.

In vitro laser irradiation of the photothermal formulations: The photothermal performance of ICG/J-ICG_DES formulations was assessed at a concentration of 25 µg. For each measurement, a 100 µL aliquot of the sample was placed into a transparent Eppendorf tube and irradiated using an infrared diode laser module (FC-D-785, CNI; λ = 785 nm, 1980 mW/cm^2). The irradiation wavelength (785 nm) was selected to coincide with the primary absorption band of monomeric ICG, ensuring efficient excitation. Although J-aggregates exhibit a red-shifted absorption maximum (~890–900 nm), they maintain appreciable absorbance at 785 nm. Moreover, ICG and J-ICG coexist in a dynamic equilibrium; thus, excitation at 785 nm effectively addresses both species and allows direct comparison of their photothermal responses under identical conditions. The irradiation protocol consisted of a 300-sec heating phase (ON) followed by a 420-sec cooling phase (OFF). Temperature variations during the ON/OFF cycles were continuously monitored using an infrared camera (FLIR E30, 25° optic, 60 Hz). The heating-cooling cycle was repeated three times, and each experiment was conducted in triplicate to ensure reproducibility. Additionally, the photothermal stability of the nanoprobes after each cycle was evaluated by UV-Vis spectroscopy.

Photothermal efficiency of J-ICG in hydrophilic/phobic DES using NIR laser: The photothermal conversion efficiency (η) of J-ICG in water and J-ICG in DES was calculated based on the steady-state temperature rise of dispersions, following the method described by Korgel et al.[2] (see section S3.4 for detailed calculations).

In vitro detection of ROS of J-ICG_DES formulations using NIR laser: The generation of ROS by J-ICG_DES formulations was evaluated using 1,3-diphenylisobenzofuran (DPBF) as an external ROS-sensitive molecule used to primarily detect singlet oxygen species ($^1O_2$). Briefly, 100 µL of J-ICG_DES dispersion (final concentration 25 µg) was

mixed with DPBF (final concentration 30 μM) in a quartz cuvette. The mixture was then irradiated at λ =785 nm using an NIR laser at room temperature. Samples were exposed to irradiation for different time intervals (0, 0.5, 1, 2, 6, 10, and 20 min) to assess the time-dependent ROS generation. Following each irradiation period, the absorption spectrum of the mixture was recorded in the range of 300-500 nm using a UV-Vis spectrophotometer (Cary 100 Bio, Agilent). The decrease in the characteristic absorbance peak of DPBF at ~415 nm was monitored as an indicator of ROS production. All measurements were performed in triplicate to ensure reproducibility. The relative decrease in DPBF absorbance over time was used to quantify the ROS generation efficiency of J-ICG_DES under NIR irradiation.

Antimicrobial tests: Staphylococcus aureus ATCC 29213, obtained from LGC Standards, was used to evaluate the photothermal antimicrobial activity of the supramolecular J-aggregates. *S. aureus* was grown in trypticase soy broth (TSB) or on trypticase soy agar (TSA) (Scharlab) at 37 °C and stored at −80 °C in the same nutrient broth supplemented with 20% glycerol until use. The overnight inoculum for the experiments was prepared by picking a colony from *S. aureus* ATCC 29213 grown on a TSA plate and inoculating it into 5 mL of TSB, followed by incubation at 37 °C for 18 h. For the antimicrobial testing, 25 μL of ICG/J-ICG_DES formulations and 25 μL of the bacterial suspension were added into sterile microtubes at a final bacterial density of 105 CFU/mL and a final ICG/J-ICG concentration of 50 μg. The samples were then irradiated with a laser for 0.5, 1, 3, or 5 min. Following irradiation, the samples were serially diluted in phosphate-buffered saline (PBS), plated on TSA, and incubated for 24 h at 37 °C to determine the number of colony-forming units (CFUs). In addition, water and DES without J-aggregates were included in this study to demonstrate the non-toxicity of the solvents. Furthermore, all samples without irradiation were processed in parallel as dark controls.

Statistical analysis: The statistical analysis was performed using GraphPad Prism 9.0 (GraphPad Software, Inc., CA, USA). P values *< 0.05, **< 0.01, and ***< 0.001 were considered significant in all analyses.

S1.2 Materials

The following chemicals were used as received: Indocyanine green (cardiogreen, Sigma-Aldrich), choline chloride–(2-hydroxyethyl)trimethyl ammonium chloride, $(CH_3)_3N(Cl)CH_2CH_2OH$ (C7017, 98%, Sigma-Aldrich), d,l-menthol–2-isopropyl-5-

methylcyclo hexanol, $C_{10}H_{20}O$ (W266507, Sigma-Aldrich), urea–NH_2CONH_2 (U5378, 99.5%, Fisher Chemicals), glycerol–1,2,3-propanetriol, $HOCH_2CH(OH)CH_2OH$ (G7893, 99%, Scharlab), pyrogallol–1,2,3-trihydroxybenzene, $C_6H_3(OH)_3$ (P0381, ≥98%, Sigma-Aldrich), glycolic Acid–hydroxyacetic acid, $HOCH_2COOH$ (124737, ≥99%, Sigma-Aldrich), tannic acid–$C_{76}H_{52}O_{46}$ (403040, 95%, Fisher Scientific), citric acid–2-hydroxy-1,2,3-propanetricarboxylic acid, $HOC(COOH)(CH_2COOH)_2$ (251275, ≥99.5%, Sigma-Aldrich), sorbitol–$C_6H_{14}O_6$ (1617000, ≥98%, Sigma-Aldrich), oleic acid–(9Z)-octadec-9-enoic acid, $CH_3(CH_2)_7CH{=}CH(CH_2)_7COOH$ (O1008, ≥99%, Sigma-Aldrich), dodecanoic acid–$CH_3(CH_2)_{10}COOH$ (L556, 98%, Sigma-Aldrich), thymol–2-isopropyl-5-methylphenol, 2-[$(CH_3)_2CH$]C_6H_3-5-(CH_3)OH (T0501, ≥98.5%, Sigma-Aldrich), lidocaine–2-diethylamino-N-(2,6dimethylphenyl), acetamide, $C_{14}H_{22}N_2O$ (L7757, ≥98%, Sigma-Aldrich), eucalyptol–1,3,3-trimethyl-2oxabicyclo[2.2.2]octane, 1,8-cineole, 1,8-epoxy-p-methane, $C_{10}H_{18}O$ (C80601, 99%, Sigma-Aldrich), ibuprofen–2-(4-Isobutylphenyl)propanoic acid, $C_{13}H_{18}O_2$ (I4883, ≥98%, Sigma-Aldrich). MilliQ water with a conductivity of 15 mΩ/cm (Merck Millipore) was used.

S2. Instrumentation

S2.1 UV/Vis spectroscopy

UV-Vis spectra were recorded using a BioTek Synergy Neo2 multi-mode reader with a 96-well plate, scanning from 300 to 999 nm at 1 nm intervals. Data analysis was performed using GEO 5 version 3.1 and GraphPad Prism version 9.3. Experimental data were processed and analyzed using GraphPad Prism 9.3.

S2.2 FTIR

Fourier-transform infrared spectroscopy with attenuated total reflectance (FTIR-ATR) was performed using a ThermoFisher Scientific Nicolet iS20 FTIR spectrometer, operating over a spectral range of 400–4000 cm^{-1}. A single drop of the sample (approximately 10 µL) was applied directly onto the ATR crystal for spectral acquisition.

S2.3 Rheometer

Viscosity measurements were performed using a TA Instruments (Waters) Discovery Hybrid HR-10 Rheometer at 25 °C. A concentric cylinder Peltier system equipped with a No. 6 spindle was employed. The samples were equilibrated for 30 seconds prior to

measurement. Shear rates ranging from 0.001 to 1000 s^{-1} were applied using a single Flow-Ramp step, with data acquisition at 1-second intervals for a total duration of 60 sec, resulting in 60 data points per sample. The Newtonian viscosity values obtained from these measurements are presented in Table S1.

S2.4 NIR Laser

The samples were exposed to irradiation using a 785 nm laser (NanoPhotontec Berlin®, model FC-D-785) positioned 1.5 cm away, delivering an intensity of 1980 mW/cm^2. The optical fiber was placed 1 cm in front of the J-ICG solution within an open Eppendorf tube. As the tube remained uncovered, minor heat exchange with the surrounding environment may have occurred, though this was considered negligible and was not accounted for in the analysis. The laser was activated for 5 min, followed by a 7-min pause, and this on/off cycle was repeated three times for each sample.

S2.5 Photothermal Camera

Temperature changes in the J-ICG solution in the presence of DES were monitored using a FLIR E53 thermal camera positioned 35 cm from the sample. This camera is capable of detecting temperatures ranging from 0 to 650 °C, with a resolution of up to 161,472 measurement points and an image capture frequency of 30 Hz. It offers a thermal sensitivity of less than 0.03 °C at 30 °C, equipped with a 42° lens. The device operates within a spectral range of 7.5 to 14.0 µm, features an f/1.3 aperture, and includes a digital zoom ranging from 1x to 4x. Depending on the lens used, the field of view is 42° × 32° (10 mm), 24° × 18° (18 mm), or 14° × 10° (29 mm). Irradiation temperature profiles were recorded as MP4 video files. For analysis, temperature data were manually extracted from the video footage and subsequently plotted using GraphPad Prism version 9.3. Temperature values obtained in each heating /cooling cycle are represented in Tables S2 and S3.

S2.6 Electron paramagnetic resonance (EPR)

Continuous-wave (CW) X-band (9.38 GHz) EPR spectra of liquid solution was collected on a Bruker EMX Plus EPR spectrometer with a 0.6 T electromagnet. The samples were prepared and transferred into a quartz EPR tube (4 mm outer diameter). For each sample, we performed a two-dimensional measurement of signal intensity

against microwave power to ensure we are in the nonsaturating regime. Fitting and simulation of the recorded EPR spectra were performed using the Xepr Bruker software, assuming a model spin Hamiltonian that consists of a sum of electron-Zeeman and electron-nuclei hyperfine interactions.

$$\hat{H} = \hat{H}_{EZ} + \hat{H}_{HF} = \mu_B \overrightarrow{B_0}^T g_k \overrightarrow{\hat{S}_k} + \sum_i \overrightarrow{\hat{S}_k}^T A_{ki} \overrightarrow{\hat{I}_i}$$

where index k refers to electron spin, index i runs over all nuclear spins, and the symbol T denotes the transpose of a vector($\overrightarrow{B_0}^T$) or vector operator ($\overrightarrow{\hat{S}_k}^T$). Note that all the measurements have been performed in liquid solution, so the $\boldsymbol{g_k}$ and $\boldsymbol{A_{ki}}$ tensors can be simplified to diagonal matrices. All fittings were performed allowing for second order corrections and anisotropic line width variations.

S3. Methods and Discussion

S3.1 FTIR Spectroscopy

DES used in this study were synthesized by combining an HBA (either ChCl or Men) with a corresponding HBD (U, Gly, Py, GlyAc, TA, CA, Sor or OleAc, DodAc, Thy, Lid, Euc, Ibu) at appropriate molar ratios. The mixtures were stirred and gently heated (typically between 60–80 °C) until a homogeneous, clear liquid was obtained, indicating complete formation of the eutectic system. FTIR spectroscopy was employed to characterize DES as demonstrated in **Figure 1b,c**. The ChCl:U displays a broad N–H stretching band around 1630 cm^{-1} and a sharper C–N stretching vibration at 1530 cm^{-1}, reflecting strong hydrogen bonding in the DES. In ChCl:Gly, a characteristic doublet appears near 2900 cm^{-1}, attributed to C–H stretching vibrations, while a weak band around 1400 cm^{-1} corresponds to O–H bending. A sharper peak between 950 and 1150 cm^{-1} indicates C–OH stretching, typical of polyol structures. In ChCl:Py, broad O–H stretching around 3200–3400 cm^{-1} is intensified due to the high hydroxyl content of Py. C=C stretching of the aromatic ring may be observed around 1600 cm^{-1}. ChCl:GlyAc shows O–H stretching near 3300 cm^{-1}, while strong C=O stretching appears around 1720 cm^{-1}, a signature of the carboxylic acid group. For ChCl:TA, broad and overlapping peaks were seen in the 3200–3400 cm^{-1} range (O–H), with complex fingerprint features between 1000–1600 cm^{-1} due to polyphenol and ester groups. ChCl:CA exhibits prominent O–H stretching near 3300 cm^{-1}, along with strong C=O vibrations at 1700–1725 cm^{-1} and C–O stretching around 1200 cm^{-1}, reflective of carboxyl and hydroxyl groups. In ChCl:Sor, the FTIR spectrum features a

strong C–OH stretching band between 950–1150 cm^{-1} and O–H bending near 1400 cm^{-1}, indicative of extensive hydrogen bonding. Across all ChCl-based DES, two consistent peaks are observed: a broad O–H stretch near 3300 cm^{-1} and a sharper band at 1500 cm^{-1}, corresponding to $(CH_3)_3N^+$ groups from ChCl.

Men:OleAc and Men:DodAc show broad O–H stretching bands around 3300 cm^{-1}, with strong C=O stretching peaks near 1700 cm^{-1}, characteristic of fatty acids. In Men:Thy, aromatic C–H stretching appears near 3000 cm^{-1}, along with O–H bands near 3200–3300 cm^{-1}. Subtle bands around 1500–1600 cm^{-1} reflect aromatic ring vibrations. Men:Lid exhibits N–H stretching near 3300 cm^{-1}, C=O stretching close to 1650–1700 cm^{-1}, and aromatic overtones in the 1500 cm^{-1} region. The Men:Euc system shows C–O–C stretching around 1100–1200 cm^{-1} due to the ether group in eucalyptol, with weaker O–H signals due to lower H-bonding. In Men:Ibu, FTIR spectra show strong C=O stretching near 1720 cm^{-1} (carboxylic acid), and aromatic C=C stretching near 1600 cm^{-1}, with minor peaks from O–H stretching above 3000 cm^{-1}.

S3.2 Viscosity Measurements

The normalized viscosity profiles (**Figure 1d,e**) provide further insight into the rheological behavior of the DES systems. The Newtonian viscosity values (n_0) of the various DES systems further highlight the significant influence of both the HBD and HBA on the rheological behavior of the formulations. For the ChCl-based systems, a wide range of viscosities was observed, from relatively low values such as 0.1146 Pa·s for ChCl:U, 0.2973 Pa·s for ChCl:Gly and ChCl:GlyAc, to significantly higher viscosities such as 28.669 Pa·s for ChCl:Sor, 45.0546 Pa·s for ChCl:TA, and reaching an extremely high viscosity of 910.3070 Pa·s for ChCl:CA. These dramatic variations can be attributed to the molecular structure and hydrogen bonding capabilities of the HBDs. For instance, polyhydroxy acids like CA and TA contain multiple hydrogen bonding sites, which lead to extensive intermolecular interactions and the formation of highly viscous, highly structured networks. In contrast, simpler molecules like urea or glycerol, which possess fewer functional groups and lower molecular weights, result in weaker hydrogen bonding networks and hence much lower viscosities.

Similarly, the menthol-based DES formulations displayed comparatively lower viscosities overall, ranging from 0.1647 Pa·s for Men:OleAc to 1.6720 Pa·s for Men:DodAc. The generally lower viscosities in these systems may be attributed to the

non-ionic nature of the mixture components and their limited capacity for forming extensive hydrogen bonding networks compared to the highly polar and ionic ChCl-based systems. The highest viscosity observed in the menthol series for Men:DodAc (1.6720 Pa·s) likely reflects the contribution of long-chain saturated fatty acids, which introduce van der Waals interactions and steric hindrance, increasing resistance to flow. The variations observed among other menthol-based DES (Men:Thy, Men:Lid, Men:Euc, Men:Ibu) further emphasize the interplay between molecular size, hydrogen bonding potential, and hydrophobic interactions in determining viscosity.

The Newtonian viscosity (n_0) was determined as the limiting viscosity at low shear rates, where the shear stress varies linearly with shear rate and viscosity remains constant:

$$\lim_{\dot{\Upsilon} \to 0} n(\dot{\Upsilon}) = n_0$$

Table S1. Newtonian Viscosity of DES.

Hydrophilic DES		Hydrophobic DES	
HBA	Newtonian viscosity, n_0 (Pa.s)	HBA	Newtonian viscosity, n_0 (Pa.s)
ChCl:U	0.1146	Men:OleAc	0.1647
ChCl:Gly	0.2972	Men:DodAc	1.6720
ChCl:Py	4.9408	Men:Thy	0.1862
ChCl:GlyAc	0.2973	Men:Lid	0.1986
ChCl:TA	45.0546	Men:Euc	0.4950
ChCl:CA	910.3070	Men:Ibu	0.3678
ChCl:Sor	28.6695		

S3.3 Near infrared-laser irradiation

S3.3.1 NIR-laser irradiation of J-ICG in the presence of hydrophilic DES

For NIR laser irradiation, the hybrid materials (100 µL; 25 µg J-ICG in DES) were placed into 0.5 mL low-binding plastic tubes. The J-ICG_DES hybrids were irradiated with a 785 nm laser at a power of 1980 mW/cm^2, as described in the main text.

Unexpected photothermal behavior was observed in the J-ICG_DES hybrids as demonstrated in the main manuscript. To investigate this further, solutions of individual components of DES were prepared and subjected to additional irradiation experiments to test the hypothesis that intramolecular hydrogen bonding in DES is primarily responsible for the altered photophysical properties of J-ICG (**Figure S1 and Table S2,S3**). During the three irradiation cycles, we observed that after each cooling phase, the temperature rose again to high levels upon subsequent irradiation. This indicates that the excess heat generated by J-ICG in the presence of DES contributes to a cumulative increase in solution temperature, as recorded by the FLIR camera. The comparison of maximum temperature rise for each DES system with its corresponding individual components highlights the significant influence of hydrogen bonding interactions on the photothermal behavior of J-ICG.

Among the ChCl-based DESs, ChCl:Gly exhibited the highest temperature elevation and photothermal conversion efficiency, likely due to its strong polyol-based hydrogen bonding network, which effectively stabilizes J-ICG aggregates through multiple hydroxyl interactions while maintaining moderate viscosity. ChCl:U also demonstrated favorable photothermal performance, where the amide functionality allows both hydrogen bond donation and acceptance, facilitating aggregate stabilization and efficient heat generation. In contrast, highly viscous systems such as ChCl:CA and ChCl:Sor, despite their abundant hydroxyl groups, showed suppressed photothermal responses, suggesting that excessively strong hydrogen bonding networks combined with limited molecular mobility hinder aggregate formation and reduce heat dissipation. A similar trend was observed in the menthol-based DESs. Men:Lid and Men:Ibu exhibited superior photothermal performance, attributed to the presence of amine and carboxyl groups that provide strong, directional hydrogen bonding interactions with menthol's hydroxyl group, promoting stable aggregate formation. Conversely, DESs formed with long-chain fatty acids such as Men:DodAc and Men:OleAc displayed lower temperature rise and efficiency, likely due to steric hindrance, weak hydrogen bonding, and a more hydrophobic microenvironment that destabilizes J-ICG aggregation. Overall, these findings emphasize that optimal hydrogen bonding strength, balanced viscosity, and sufficient polarity are essential for promoting stable J-ICG aggregation and enhancing photothermal conversion efficiency, while excessive hydrophobicity, steric hindrance, or overly rigid hydrogen bonding networks can significantly impair photothermal performance.

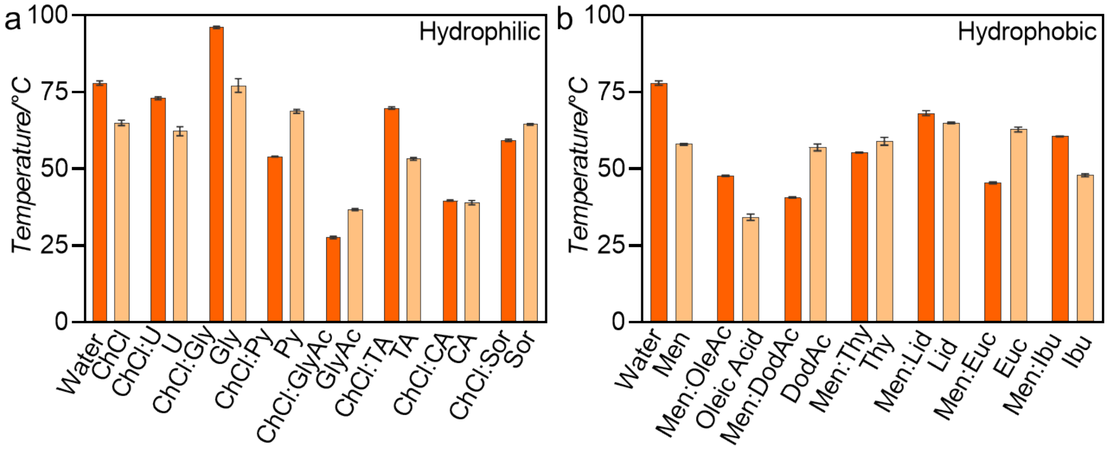

Figure S1. Maximum (T_{max}) temperatures recorded during heating-cooling cycles for J-ICG in presence of (a) ChCl-, and (b) Men-based formulations, each combined with individual components of DES: ChCl, U, Gly, Py, GlyAc, TA, CA, Sor; Men, OleAc, DodAc, Thy, Lid, Euc, and Ibu. ChCl:U, ChCl:Gly, ChCl:TA, Men:OleAc, Men:Lid, Men:Ibu shows more photothermal efficiency than individual components.

Table S2. Temperature profiles (°C) during heating-cooling cycles for water and hydrophilic ChCl-based formulations containing various HBD. T_{max} indicates the maximum temperature reached during the heating phase for each formulation.

	Temperature (°C)							
Time (min)	Water	ChCl:U	ChCl:Gly	ChCl:Py	ChCl:GlyAc	ChCl:TA	ChCl:CA	ChCl:Sor
0	26.9	30.4	28.9	24.1	24.0	24.7	31.2	25.4
5	78.5	72.7	96.4	52.4	27.9	69.6	39.5	59.6
12	26.2	36.5	26.9	25.9	23.9	27.0	26.7	27.3
17	77.5	44.5	84.6	44.6	27.3	64.2	37.4	57.2
24	26.0	35.1	27.4	25.5	25.1	27.3	26.5	27.3
29	69.6	44.0	73.1	39.0	27.1	58.6	36.0	52.6
36	26.5	25.9	27.5	25.9	24.6	26.5	26.1	27.1
T_{max}	78.5 ± 2.8	72.7 ± 9.5	96.4 ± 6.7	53.9 ± 3.5	27.9 ± 0.2	69.6 ± 3.2	39.5 ± 1.1	59.6 ± 2.1

Table S3. Temperature profiles (°C) during heating-cooling cycles for water and menthol-based formulations containing various components: OleAc, DodAc, Thy, Lid, Euc, and Ibu.

	Temperature (°C)						
Time (min)	Water	Men:OleAc	Men:DodAc	Men:Thy	Men:Lid	Men:Euc	Men:Ibu
0	26.9	25.9	25.9	25.6	25.8	25.8	27.9
5	78.5	45.9	40.5	54.8	68.7	45.7	57.7
12	26.2	25.7	25.4	25.7	26.2	26.0	26.1
17	77.5	41.5	39.1	54.3	65.2	42.3	50.4
24	26.0	25.7	25.4	25.5	25.9	25.7	26.2
29	69.6	39.8	36.4	53.3	63.6	38.7	43.7

36	26.5	25.9	25.9	25.6	25.8	25.8	25.9
T_{max}	78.5 ± 2.8	47.9 ± 1.9	40.5 ± 0.9	55.2 ± 0.4	68.7 ± 1.5	46.2 ± 1.7	60.5 ± 3.8

S3.3.2 NIR-laser irradiation of J-ICG in the presence of individual components of DES

Table S4. Temperature profiles (°C) during heating-cooling cycles for J-ICG in the presence of individual components of hydrophilic DES.

	Temperature (°C)							
Time (min)	ChCl	U	Gly	Py	GlyAc	TA	CA	Sor
0	26.9	26.1	28.5	26.0	28.0	25.1	26.2	26.4
5	64.4	63.3	74.6	61.1	37.0	52.9	39.5	64.7
12	28.0	27.4	28.7	27.7	25.7	26.9	26.4	28.4
17	55.8	58.5	78.7	68.3	34.5	47.4	39.0	61.2
24	27.4	27.9	29.7	28.0	25.6	26.7	26.0	28.2
29	48.9	53.3	75.5	44.5	33.5	44.5	38.8	56.0
36	27.1	27.7	29.5	26.4	25.7	26.5	26.2	27.5
T_{max}	64.4 ± 4.9	63.3 ± 2.8	78.7 ± 1.2	68.3 ± 7.1	37.0 ± 1.1	52.9 ± 2.5	39.5 ± 0.2	64.7 ± 2.5

Table S5. Temperature profiles (°C) during heating-cooling cycles for J-ICG in the presence of individual components of hydrophobic DES.

	Temperature (°C)						
Time (min)	Men	OleAc	DodAc	Thy	Lid	Euc	Ibu
0	27.8	28.0	25.0	25.7	24.3	26.4	24.8
5	57.0	35.0	56.0	58.5	65.1	62.9	47.6
12	27.4	25.9	27.9	27.7	28.2	28.3	27.2
17	48.9	33.5	50.0	52.8	51.4	53.1	46.8
24	27.0	25.9	27.3	27.0	27.4	27.6	27.2
29	47.0	31.5	47.8	49.8	43.5	49.6	43.3
36	26.6	25.4	27.2	27.0	26.9	27.4	26.7
T_{max}	58.2 ± 2.8	35.0 ± 1.0	57.8 ± 2.5	59.9 ± 2.6	65.2 ± 6.3	63.4 ± 3.5	47.6 ± 1.3

S3.3.3 NIR-laser irradiation of ICG monomer in the presence of DES

We examined the photothermal response of ICG monomer in the presence of different DES, using them as controls (**Figure S2**). As expected, laser irradiation at 785 nm did not result in efficient photothermal conversion in these systems. The maximum temperatures achieved were modest, ranging from 24.0 to 46.8 °C for hydrophilic DES and 30.5 to 62.5 °C for hydrophobic DES. This limited heating effect highlights the weak photothermal conversion capacity of the ICG monomer under these conditions.

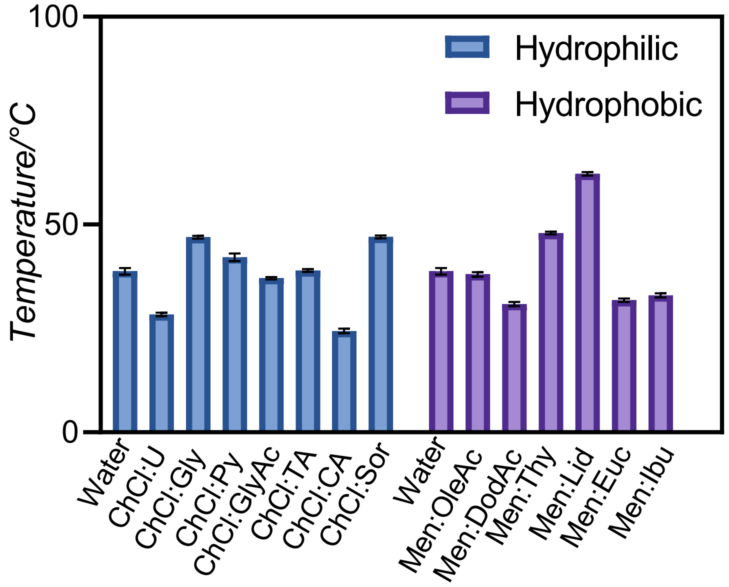

Figure S2. Maximum (T_{max}) temperatures recorded during heating-cooling cycles for ICG monomer in the presence of (a) ChCl-, and (b) Men-based formulations.

S3.3.4 UV-visible spectra of J-ICG in presence of DES

UV–Vis absorption spectra of J-ICG recorded across the 550–950 nm range for all hydrophilic and hydrophobic DES formulations (**Figure S3 and S4**, respectively). All formulations were prepared at a 70:30 v/v DES:water ratio for absorption spectroscopy measurements, a composition that preserves the intermolecular hydrogen bonding network while ensuring sufficient optical clarity for spectroscopic analysis.[3,4] For the hydrophilic DES systems (**Figure S3**), ChCl:U, ChCl:Gly, ChCl:TA, ChCl:CA, and ChCl:Sor, displays a well-defined J-aggregate absorption band at ~890–900 nm. Notable exceptions are ChCl:Py, which exhibits anomalously high broad background absorption attributable to pyrogallol itself, and ChCl:GlyAc, which shows very low overall absorbance with a poorly resolved J-band. For the hydrophobic menthol-based DES systems (**Figure S4**), all formulations exhibit remarkably consistent and closely overlapping spectra with a clearly resolved J-aggregate band at ~890–900 nm and uniformly low absorbance at 785 nm.

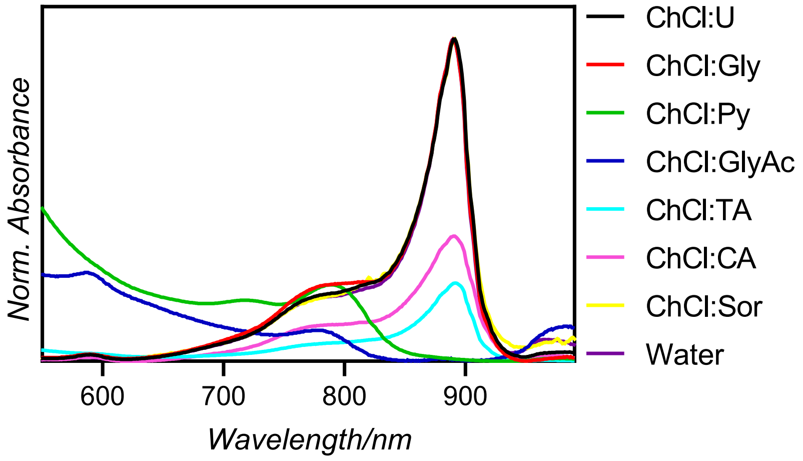

Figure S3. UV-Visible spectra of J-ICG in the presence of hydrophilic DES.

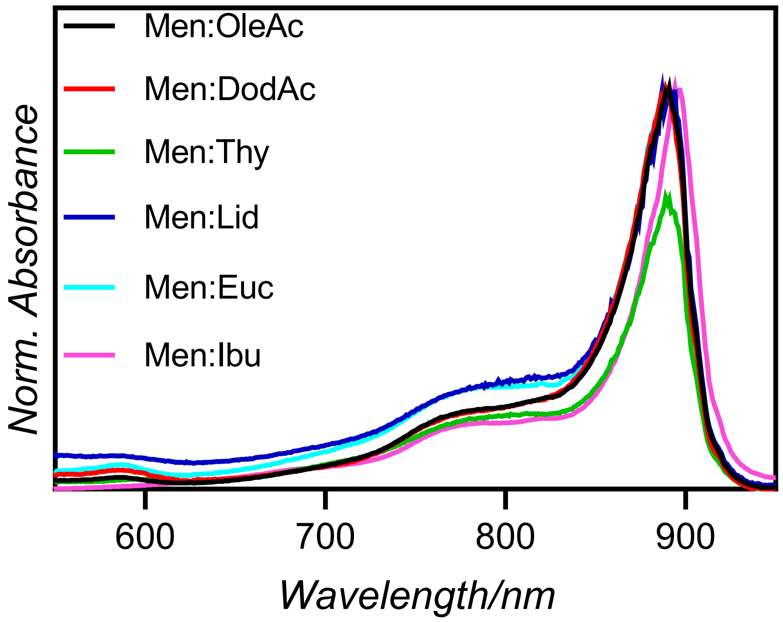

Figure S4. UV-Visible spectra of J-ICG in the presence of hydrophobic DES.

S3.3.5 Thermal and biological stability of J-ICG in the presence of DES

To assess the stability of J-ICG aggregates under conditions representative of photothermal irradiation, time-resolved UV–Vis absorption spectra were recorded for J-ICG in all DES formulations maintained at 95°C over an 8-minute (**Figure S5-S7**). A progressive and gradual attenuation of the J-aggregate band at ~890–900 nm is observed with increasing time.

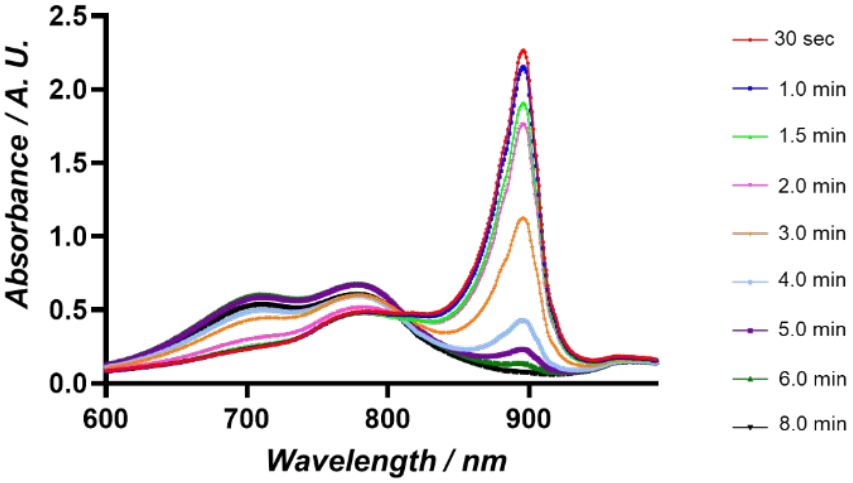

Figure S5. Thermal stability of J-ICG in water.

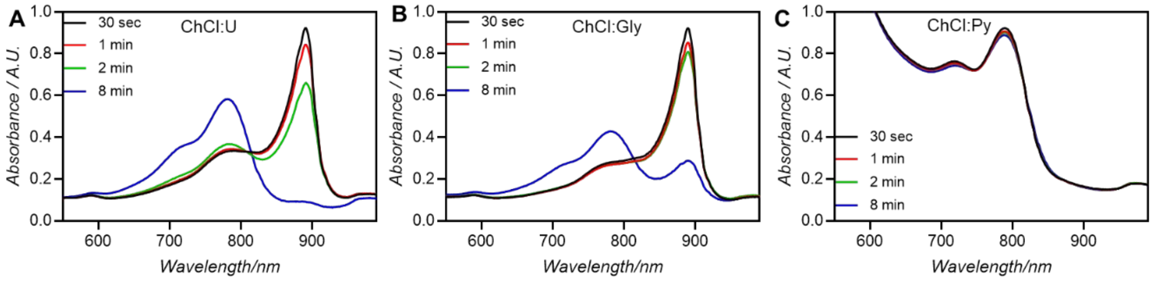

Figure S6. Thermal stability of J-ICG in the presence of A) ChCl:U, B) ChCl:Gly, and C) ChCl:Py.

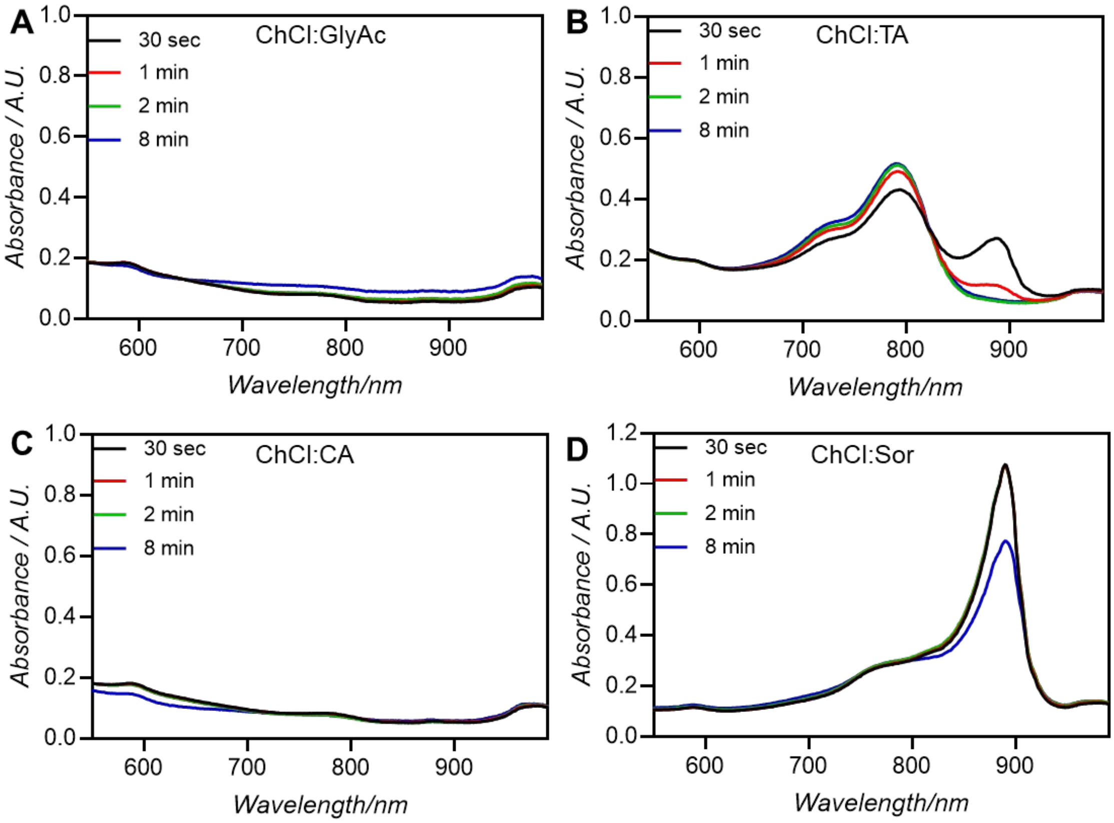

Figure S7. Thermal stability of J-ICG in the presence of A) ChCl:GlyAc, B) ChCl:TA, C) ChCl:CA, and ChCl:Sor.

The biological stability of J-ICG_DES (ChCl:Gly) was further evaluated by incubation in 100% fetal bovine serum (FBS), Dulbecco's Modified Eagle Medium (DMEM), and DMEM supplemented with FBS for 1 hour (**Figure S8**). The J-aggregate band at ~890–900 nm is largely retained across all biological media, with only a modest gradual reduction in intensity attributable to progressive exchange of DES components with proteins and electrolytes.

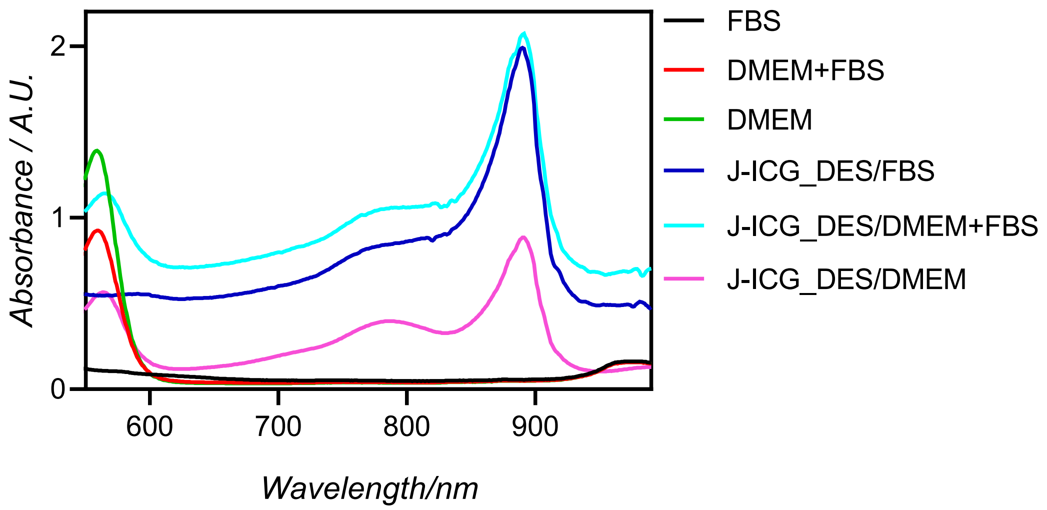

Figure S8. Stability of J-ICG_DES in biological media. UV–Vis spectra of J-ICG_DES (ChCl:Gly) in FBS, DMEM, and DMEM + FBS show retention of the J-aggregate band (~890–900 nm).

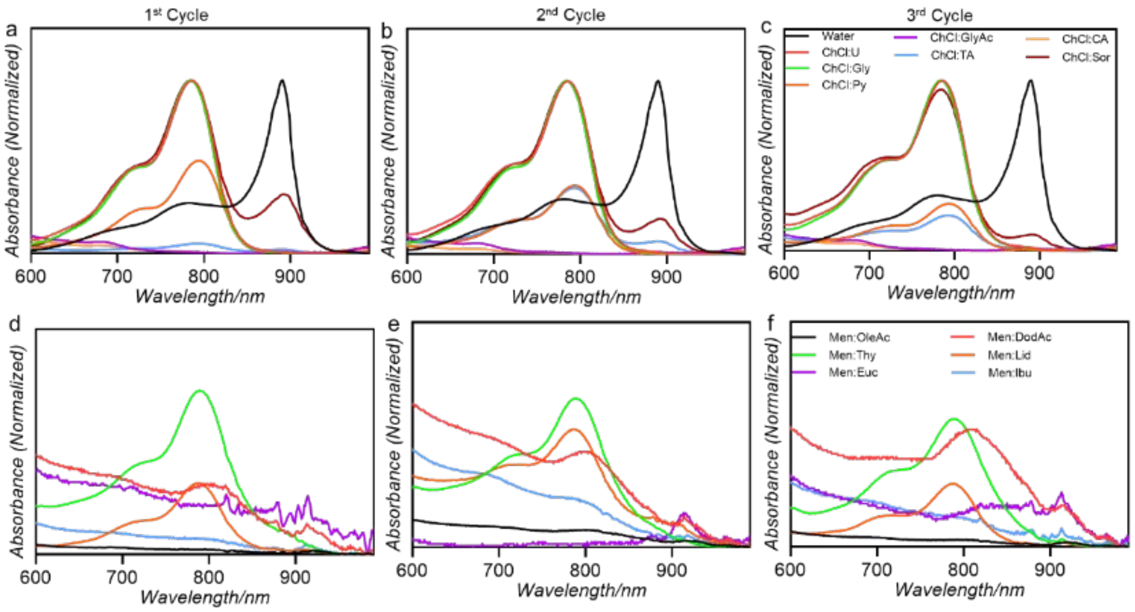

Figure S9. UV-Vis absorption spectra of J-ICG formulations after irradiation and heating-cooling cycles. (a–c) Spectra for ChCl-, (d–f) Men-based systems containing respective HBD components. Spectral variations reflect the changes in J-ICG stability and aggregation state induced by individual additives after thermal cycling and irradiation.

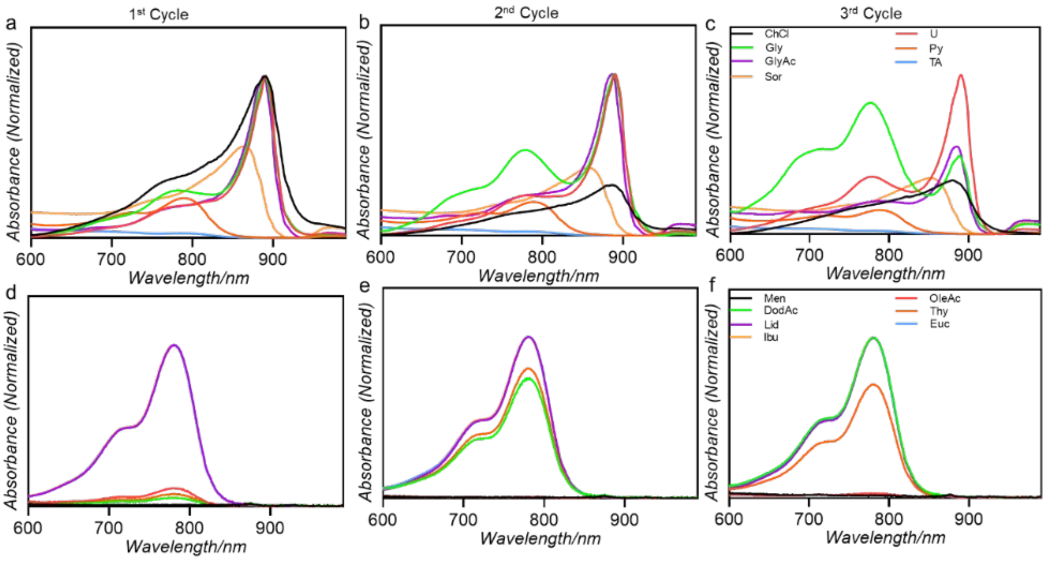

Figure S10. UV-Vis absorption spectra of J-ICG formulations after irradiation and heating-cooling cycles of all individual components. (a–c) Spectra for ChCl-, (d–f) Men-based systems.

S3.3.6 FTIR Spectra of J-ICG_DES formulation

To understand differential hydrogen bonding interactions between DES formulations and J-ICG, we performed ATR-FTIR analysis of J-ICG in water and in all hydrophilic DES systems (**Figure S11**) as these systems demonstrated consistently superior photothermal performance compared to their hydrophobic counterparts, attributed to their greater ability to interact. Systematic shifts and broadening of C–O stretching bands (~900–1200 cm^{-1}) are observed across DES formulations relative to J-ICG in water, confirming the establishment of hydrogen bonding interactions between the DES matrix and the ICG chromophore. Notably, ChCl:Sor induces the most pronounced shift compared to ChChl:Gly, indicating that sorbitol's multiple hydroxyl groups engage in the strong overall hydrogen bonding interactions with the J-ICG.

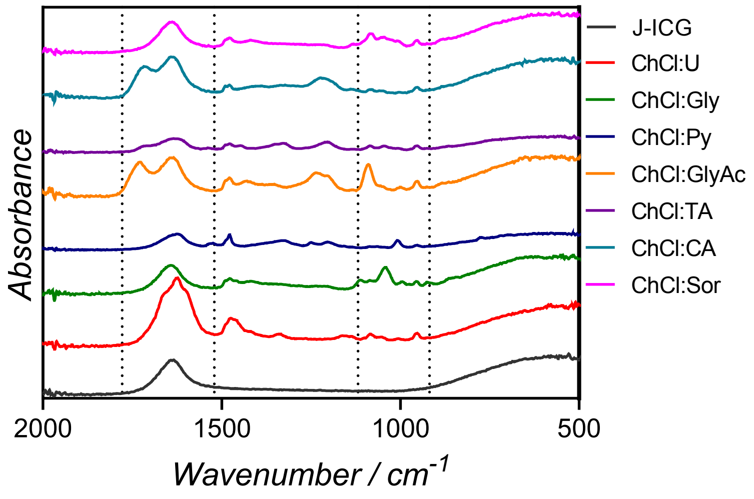

Figure S11. FTIR spectra of J-ICG in the presence of DES.

S3.4 Calculation of photothermal conversion efficiency (η)

$$\eta = (hA(T_{max} - T_{surr}) - Q_{in,\ surr})/(I(1 - 10^{A_\lambda}) \qquad \text{(Eq. S1)}$$

Where h is the heat transfer coefficient, A is the surface area of the container, T_{max} is the maximum steady-state temperature, T_{surr} is the ambient surrounding temperature, I is the laser power (1980 mW/cm^2), A_λ is the absorbance at the excitation wavelength of 785 nm (1.68 AU). $Q_{in,surr}$ is the heat input due to light absorption by the water medium (3.25 mW). The quantity hA was calculated as follows:

$$hA = m_w\ Cp_w /\ \tau_s \qquad \text{(Eq. S2)}$$

Where m_w and Cp_w are the mass and specific heat capacity of the water medium, and τ_S was calculated by measuring the rate of temperature drop after removing the laser source, from the following equations:

$$t = -\ \tau_s\ ln\theta \qquad \text{(Eq. S3)}$$

$$\theta = T - T_{surr}\ /\ T_{max} - T_{surr} \qquad \text{(Eq. S4)}$$

S3.4.1 Photothermal conversion efficiency of J-ICG in the presence of hydrophilic DES

Table S6. Photothermal parameters of J-ICG (25 µg in 100 µL) in hydrophilic DES under NIR laser irradiation (785 nm, 1980 mW/cm^2). The calculated heat transfer coefficient (hA) and photothermal conversion efficiency (η) are based on experimental heating-cooling data.

**DES**	**hA (mW/K)**	η(%)
Water	4.43	15.4
ChCl:U	4.10	12.6
ChCl:Gly	4.68	21.7

ChCl:Py	3.85	7.1
ChCl:GlyAc	-	-
ChCl:TA	5.60	16.2
ChCl:CA	3.80	3.4
ChCl:Sor	4.05	9.0

S3.4.2 Photothermal conversion efficiency of J-ICG in the presence of hydrophobic DES

Table S7. Photothermal parameters of J-ICG (25 µg in 100 µL) in hydrophobic DES under NIR laser irradiation (785 nm, 1980 mW/cm^2). The calculated heat transfer coefficient (hA) and photothermal conversion efficiency (η) are based on experimental heating-cooling data.

DES	hA (mW/K)	η%
Men:OleAc	5.48	8.0
Men:DodAc	5.77	5.7
Men:Thy	4.97	9.6
Men:Lid	4.47	12.6
Men:Euc	5.14	6.9
Men:Ibu	4.22	9.5

S4. *In vitro* ROS detection and antimicrobial activity

Singlet-oxygen generation was quantified by monitoring DPBF bleaching at 415 nm (DPBF = 30 µM; pathlength = 1 cm; total volume = 1 mL; **Figure S12**). The amount of $^1O_2$ produced was calculated from the decrease in DPBF, which reacts approximately 1:1 with $^1O_2$ until the probe is consumed. Under continuous irradiation, the decay of DPBF absorbance at 415 nm gives a linear semilog plot ln(A_0/A_t) vs. t, confirming pseudo-first-order kinetics for the J-ICG_DES–sensitized reaction. For the reaction of DPBF with $^1O_2$ in steady state, the disappearance rate of the substrate is: $\frac{d[DPBF]}{dt} = -k_q[O_2^1][\mathrm{DPBF}] = -k_{\{obs\}}[DPBF]$ (Eq. S5)

$$\ln\left(\frac{[DPBF]_t}{[DPBF]_0}\right) = -k_{\{obs\}}.t \quad \text{(Eq. S6)}$$

$$\ln\left(\frac{[DPBF]_0}{[DPBF]_t}\right) = k_{\{obs\}}.t \quad \text{(Eq. S7)}$$

Replacing DPBF concentration by corresponding absorbance at 415 nm:

$$A = \varepsilon.b.[DPBF] \quad \text{(Eq. S8)}$$

$$ln\frac{[A]_0}{[A]_t} = k_{\{obs\}}.t \quad \text{(Eq. S9)}$$

The cumulative $^1O_2$ captured was ~0.9, 1.3, 3.8, 8.7, 12.1, and ~17.7 μM at 0.5, 1, 2, 6, 10, and 20 min, respectively.

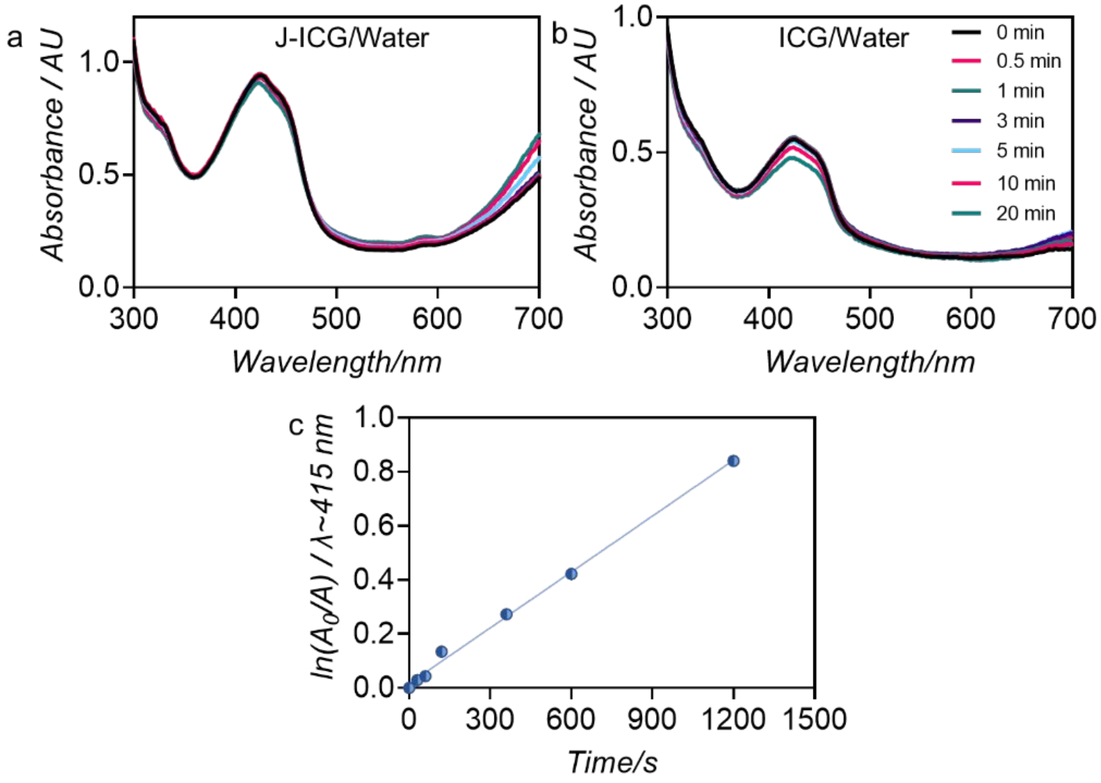

Figure S12. Detection of reactive oxygen species (ROS) generation by using 1,3-diphenylisobenzofuran (DPBF) as an external probe. (a) J-ICG and (b) ICG in water. Pseudo first-order plots for the consumption of DPBF photosensitized by J-ICG_DES in air-equilibrated solution (λ_{irr}=785 nm, 1980 mW/cm^2).

In vitro hydroxyl radicals detection.

In order to investigate the possible generation of hydroxyl radicals, we performed terephthalic acid (TA) fluorescence assays under our experimental conditions. Control experiments including TA_ChCl:Gly, TA+J-ICG_ChCl:Gly (light and dark) were systematically evaluated, along with appropriate blank corrections (**Figure S13**). However, no appreciable fluorescence signal corresponding to 2-hydroxyterephthalic acid (λ_{em} ~ 425 nm) was observed upon NIR irradiation. These results suggest that hydroxyl radical generation is negligible in our system under the studied conditions. Accordingly, the photodynamic activity of the J-aggregate system is likely dominated by singlet oxygen, with limited contribution from hydroxyl radical-mediated pathways.

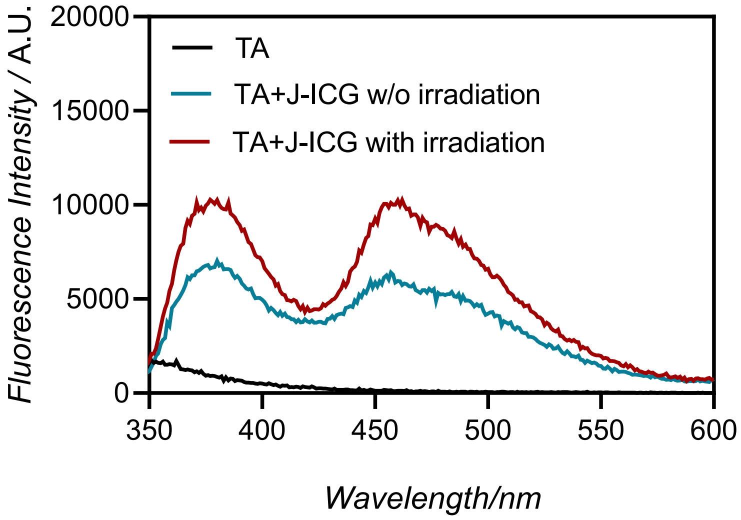

Figure S13. Assessment of hydroxyl radical generation using terephthalic acid (TA). Fluorescence spectra of TA after irradiation show no detectable emission at λ_{em} ~425 nm compared to the control, indicating the absence of hydroxyl radical (•OH) formation.

Electron spin resonance.

Furthermore, a spin-trapping experiment using the spin trap PBN (phenyl *N*-t-butylnitrone) was performed to detect the formation of radical species during irradiation by cw-EPR spectroscopic analysis. Therefore, J-ICG_DES was mixed with PBN in a 1:20 ratio. A cw-EPR spectrum before and after irradiation of the sample for 20 min with a NIR laser was recorded, as shown in **Figure S14**. An EPR signal could only be detected after irradiation of the sample. A simulation of the obtained spectrum could be referred to a main paramagnetic species with the hyperfine coupling parameters A_N = 15.89 G and A_H = 3.52. These results can be assigned to the formation of $^{\bullet}OOH$ or related species. Additionally, attempts were made to record EPR spectra without the use of a spin trap; however, no detectable signal was observed, likely due to the low steady-state concentration and short lifetime of transient radicals, as well as experimental limitations associated with irradiation within the capillary tube setup.

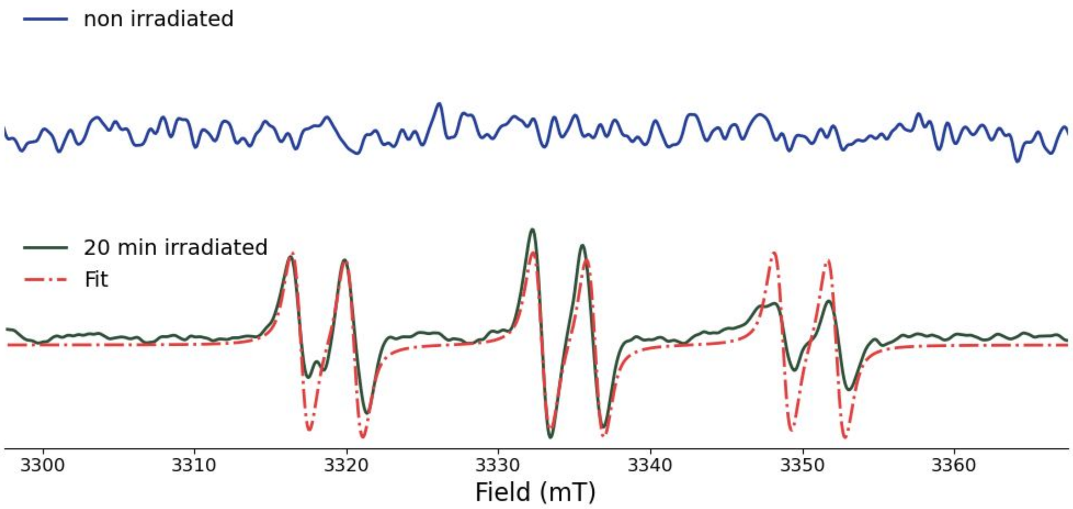

Figure S14. Comparison of the cw X-band EPR spectra of a mixture of J-ICG_DES (ChCl:Gly) and the spintrap PBN (1:20 ratio). The upper panel shows the spectrum obtained before irradiation. The lower panel shows the spectrum after irradiation (green) with the corresponding spectral fitting (red, dashed).

$$\% \text{ Reduction}=(1-N/N_0) \times 100 \qquad \text{(Eq. S10)}$$

N_0-Control; N-Treated sample

$$\text{CFU/mL}= (\text{colonies counted} \times \text{dilution factor})/\text{volume plated (mL)} \qquad \text{(Eq. S11)}$$

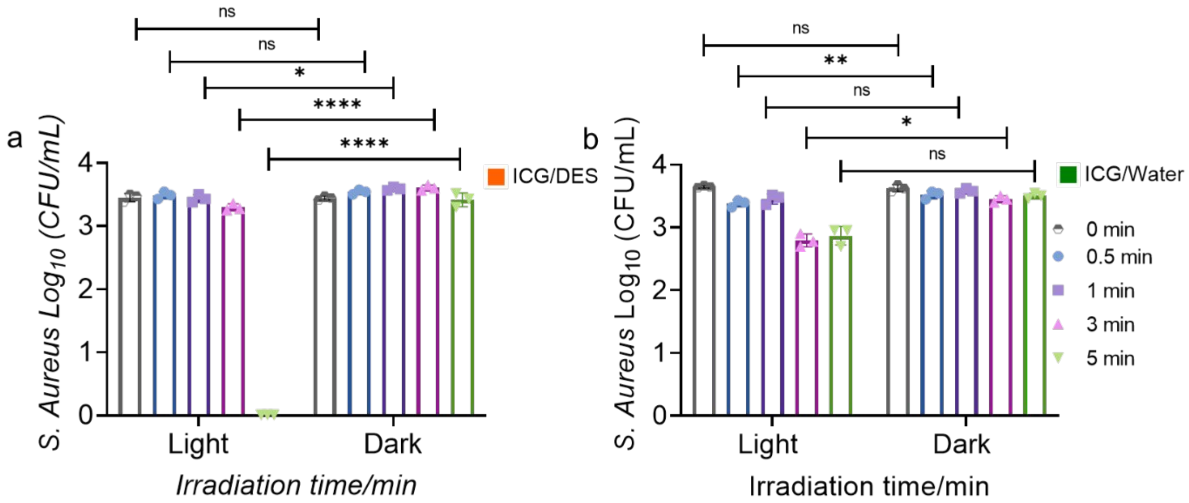

Figure S15. *S. aureus* cells incubated in (a) ICG/DES, and (b) ICG/water before and after laser irradiation (λ=785 nm; 1920 mW cm^{-2}). Bacterial survival expressed as log CFU/mL at varying irradiation times under laser ON (orange bars) and laser OFF (beige bars) conditions. Results are expressed as the mean ± standard deviation from two independent experiments (n = 3). Statistical analysis was performed using two-way ANOVA followed by Bonferroni's multiple comparison test. Asterisks denote significant differences (*p < 0.05, **p < 0.01, ***p < 0.001, ****p < 0.0001).

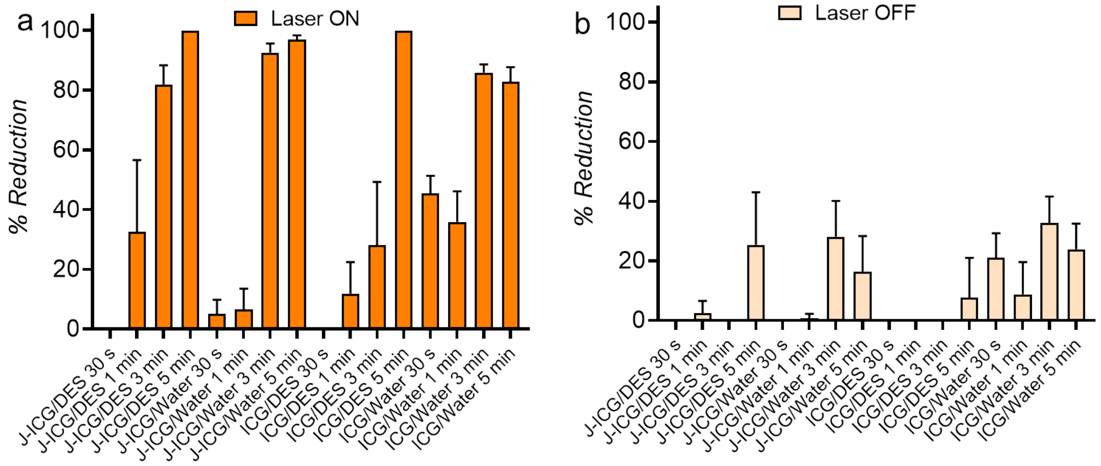

Figure S16. Antibacterial activity expressed as percentage reduction in bacterial colony count using the colony count method under two conditions: (a) laser ON (orange bars), and (b) laser OFF (beige bars).

References

1. Bhavane, R.; Starosolski, Z.; Stupin, I.; Ghaghada, K. B.; Annapragada, A. NIR-II fluorescence imaging using indocyanine green nanoparticles, Sci. Rep., **2018**, *8*, 14455.

2. Zhao, B. Y.; Xu, P.; Yang, F. X.; Wu, H.; Zong, M. H.; Lou, W. Y. Biocompatible Deep Eutectic Solvents Based on Choline Chloride: Characterization and Application to the Extraction of Rutin from Sophora japonica. ACS Sustainable Chem. Eng.*,* **2015**, *3 (11)*, 2746–2755.
